# Study of deep learning cues for cross linguistic part of speech tagging in English– Malayalam code-mixed data

**DOI:** 10.3389/fdata.2026.1888885

**Published:** 2026-08-28

**Authors:** Parvathy Padmakumar, Shreya S. Nair, A. K. Prajisha, S. Thara

**Affiliations:** Department of Computer Science and Engineering, Amrita School of Computing, Amrita Vishwa Vidyapeetham, Amritapuri, Kollam, India

**Keywords:** BiLSTM, code-mixed NLP, CRF, low-resource languages, Malayalam, part-of-speech tagging, transformer models

## Abstract

**Introduction:**

Part Of Speech (POS) tagging is a fundamental task in Natural Language Processing (NLP) that assigns grammatical labels to words in a sentence. Code mixed text, which entails switching between two or more languages within a single conversation or a sentence, presents challenges for POS tagging. This investigation entailed a comprehensive study of deep learning approaches for cross linguistic POS tagging, focused on English Malayalam code mixed data prevalent on social media platforms. The study was carried out on linguistically complex and varied English Malayalam code mixed text from social media platforms with informal spellings, language switching, transliteration, slang, and unclear grammatical boundaries, reflecting the characteristics of informal online communication. We provide the first structured analysis of micro macro divergence and rare class behavior in English Malayalam code mixed POS tagging.

**Methods:**

We evaluated 14 state of the art model configurations that span traditional sequence labeling approaches and multilingual transformer architectures. Models were compared using standard performance metrics prevalent in the domain of data science, supplemented by normalized confusion matrices, error prone tag identification and micro-macro F1 gap analysis.

**Results:**

Our results showed that CRF (No Lang) emerged as the most balanced model overall on macro F1 (all classes) of 0.8170, while (BiLSTM + CRF) achieved the highest macro F1 (seen classes) of 0.8831, precision of 0.9167, and recall of 0.875, though this reflects strong performance concentrated on frequent tag classes rather than balanced coverage across the full tag set. Notably, the pretrained multilingual transformers (mBERT, MuRIL), despite prior exposure to Malayalam during pretraining, were outperformed on several key metrics by CRF and BiLSTM models trained directly on the code mixed dataset.

**Discussion:**

This finding was contrary to our expectation that existing multilingual knowledge would translate into a clear advantage on this task and merits further investigation.

## 1 Introduction

Code mixing is the linguistic practice where multilingual speakers sport their familiarity with more than one language in their communicative interactions, frequently alternating at the word, phrase, or sentence level. Code switching is when a speaker or writer alternates between two or more languages within a single conversation, sentence, or even a phrase. Both terms are interchangeably used most of the time, but theoretically, code-mixing is intra sentential, while code switching is inter sentential. For clarity, we use “code-mixing” throughout to refer to the intra sentential blending of English and Malayalam that constitutes our dataset and the phenomenon under study. We use “code-switching” only when discussing prior work that specifically frames the phenomenon in those terms. The code mixing practice is flourishing in social media platforms, to the detriment of Natural Language Processing (NLP) models which need to instantaneously switch between languages. In addition, the models are expected to ensure that the resultant interpretation is correct and linguistically meaningful. The exponential rise in adoption of code mixed sentences on social media platforms, have afforded a corpus of formidable datasets for NLP applications Chatterjee et al., 2022.

Language-pair names (e.g., “English–Malayalam” vs. “Malayalam–English,” “Hindi–English” vs. “English vs. Hindi”) are used interchangeably throughout this paper and in the cited literature; the order carries no linguistic significance and follows each source's own convention.

India is one of the world's most linguistically enriched nations, with hordes of unexplored regional languages. Malayalam, native language in the state of Kerala in South India, spoken by approximately 38 million people, is routinely code-mixed with English in digital media. Students and professionals in Kerala coolly resort to code mixing, to communicate on social media platforms, with a free and ambiguous use of English and Malayalam, not easily captured by NLP models.

Part Of Speech (POS) tagging and language identification (LID) are the fundamental NLP tasks, which serve as foundational steps in downstream applications of sentiment analysis and machine translation Thara and Poornachandran, 2021. Despite commendable advances in this arena, current architectures—including standard monolingual POS taggers and rule-based systems, along with multilingual transformer models like XLM-Robert—are constrained by inaccurate handling of multilingual and code mixed expressions; traditional monolingual NLP models fails to adapt on account of a number of factors:

- Words from one language are used in contexts where the other language should have dominated.
- Noncompliant grammatical rules of either or both languages.
- Increasing complexity when two or more words can be used together to create a single word, in languages like Malayalam.

Recent advances in Deep Learning and Transformer architectures, which generate different contextual word embeddings, for the same word, have shown strong potential for code mixed NLP tasks Jain et al., 2026; Munyao, 2025. Bidirectional Long Short Term Memory (BiLSTM) networks are widely adopted for sequence labeling, galvanized by their ability to utilize past and future tokens for the capture of contextual information. Recent developments of Transformer based models like Multilingual Bidirectional Encoder Representations (MuRIL), Khanuja et al. (2020), and RoBERTa, Conneau et al. (2020), have shown awesome performance across multiple languages when pretrained on large multilingual corpora and cross lingual representations.

Existing work focus on model performance, but lack systematic analysis of class imbalance ad cross lingual error behavior in code mixed POS tagging. Exuberant ongoing studies also focus on high resource language pairings of English Spanish, English Hindi, and English German, leaving aside code mixing of English with low-resource languages like Malayalam. Appraisals of code mixed model performance have been limited to overall performance have been limited to overall performance to the exclusion of systematic analysis of language behavior, error patterns, language transition boundaries, and proportion of code mixing in a sentence. Comparative performance analysis of BiLSTM versus Transformer architectures for POS labeling of code mixed English Malayalam has not seen the light of day.

This paper explored the potential of numerous deep learning approaches for cross linguistic POS tagging on code mixed English Malayalam. The model selection was designed around a central comparison: architectures that learn code-mixed representations directly from the target dataset (CRF and BiLSTM, in their various feature/regularization/gazetteer configurations) versus architectures that arrive with existing multilingual pretraining on Malayalam and English (mBERT, pretrained on 104 languages including Malayalam, and MuRIL, pretrained specifically on 17 Indian languages in transliterated form). This contrast tests whether prior multilingual knowledge transfers effectively to the specific challenge of English–Malayalam code-mixed POS tagging, or whether task-specific learning form the code-mixed data itself is more valuable given the absence of code-mixed text in either model's pretraining corpus. The 14 model configurations represent all variants evaluated in this study. The evaluated models include six CRF variants, four BiLSTM-based variants, two mBERT variants, and two MuRIL variants. They were selected to compare the major sequence-labeling approaches for English–Malayalam code-mixed POS tagging, including CRF-based, BiLSTM-based, and multilingual transformer models on the following performance metrics:

- Q1. Language-wise performance: how does the model performance differ between English and Malayalam tokens?
- Q2. Impact of language balance: does the balance of tokens in the dataset or models explain observed performance differences?
- Q3. Robustness across frequency: how stable is each model when handling common versus rare POS categories?
- Q4. Misclassification analysis: Which POS tags are most frequently misclassified across models?
- Q5. Per-class performance variation: how does performance vary across high-frequency POS tags for each model?
- Q6. Model comparison and selection: how do CRF, BiLSTM, and transformer-based models compare across accuracy, precision, recall, macro F1 (seen and all classes), and weighted F1—and what factors determine the best performing model?

## 2 Related work

Code mixing and code switching in multilingual communities have been a subject of keen interest for several decades. As an offshoot for NLP, early computational work on code mixed NLP was focused primarily on LID at the token level, a prerequisite for most downstream tasks, besides POS tagging. Solorio and Liu (2008) were among the first to address POS tagging for code switched Spanish English data, demonstrating that LID features could augment tagging accuracy. Their work established the foundation for treating code mixed text as a distinct linguistic phenomenon mandating sophisticated models rather than simple application of monolingual taggers.

For Indian languages specially, the Task on Code Mixed Social Media Text, Jamatia and Das (2016), organized as part of ICON (International Conference on Natural Language Processing, India) and EMNLP (Empirical Methods in Natural Language Processing, India) shared tasks, provided standardized datasets and evaluation benchmarks for Hindi English and other language pairs referenced here. Vyas et al. (2014) introduced a grammatical framework for LID and POS tagging in Hindi English code mixed data from Facebook, throwing light on the intricacy of the task and the inadequacy of existing monolingual tools. Sequeira et al. (2015) built up on Vyas's concept by investigating Conditional Random Field based models that included language specific features for the same language pair, showing that carefully designed language features could outperform baseline models relying solely on word identity.

Malayalam English code mixing has received relatively less attention in the NLP domain. Malayalam is a rich Dravidian language with a complex structure comprised of combining and additive features, which kick in arduous tasks for the POS tagging of Romanized or transliterated social media text Premjith et al., 2024; Sreelakshmi et al., 2024. Investigators have observed that regular NLP tools do not work well on Malayalam text, as they are designed for authentic Malayalam script and incapable of handling the informal spelling variations conspicuous on social media platforms like Twitter and WhatsApp Harikrishnan and Bindu, 2024; Gopalakrishnan et al., 2025.

The introduction of pretrained multilingual language models (e.g., MuRIL and mBERT) has significantly altered the landscape of NLP for low-resource and code mixed languages. Devlin et al. (2019) introduced BERT, a bidirectional transformer-based language model. Its multilingual variant (mBERT), trained on 104 languages using a shared WordPiece vocabulary, facilitates cross-lingual transfer in zero-shot and few-shot settings. Khanuja et al. (2020) introduced MuRIL, a BERT-based model pretrained on 17 Indian Languages and their transliterated forms, which was shown to exceeded mBERT on several Indian language benchmarks. However, the performance of these models on code mixed Malayalam English POS tagging was not thoroughly evaluated alongside traditional sequence labeling approaches. Also, prior works do not evaluate rare-class robustness; most studies report only accuracy, masking imbalance issues.

Prior work on CRF based POS tagging for code mixed Indian languages has established that features such as character *n* grams, suffix and prefix patterns and language identity tags contribute meaningfully to model performance Gautam, 2022; Nair and Thushara, 2025. Gazetteer lists of named entities and location names were used in a few systems, despite their benefit's dependency on the nature of the dataset. BiLSTM based models have surpassed CRFs on several sequence labeling tasks by autonomous learning of contextual representations. The combination of BiLSTM with a CRF output layer, proposed by Lample et al. (2016) for named entity recognition, is a widely used architecture for POS tagging, adapted by several researchers for code mixed settings. FastText embeddings, which represent words using character n gram sub word information, have been widely explored for morphologically rich languages and are better suited to capture Malayalam morphology than word-level embeddings.

Despite the body of work in POS tagging, several gaps remain unaddressed in the literature:

- No systematic English–Malayalam POS resource comparable in scale or annotation rigor to the Hindi-English shared-task datasets exists, so tagging performance for this pair has not been benchmarked under standardized conditions.
- Prior CRF, BiLSTM, and transformer studies on Indian-language code-mixing are evaluated independently rather than compared side-by-side on the same dataset and splits, making cross-architecture claims (e.g. “transformers outperform CRFs”) difficult to verify for Malayalam specifically.
- Class imbalance is acknowledged in prior code-mixed POS work but rarely analyzed at the level of individual rare tags; aggregate macro F1 is typically reported without checking whether rare classes were predicted at all.
- Malayalam-specific sub word embeddings have not been evaluated in this line of work; existing FastText-based studies on Indian code-mixing typically default to generic multilingual or Hindi vectors, leaving open whether Malayalam-trained sub word embeddings would close the gap with transformer models.
- Language-level error analysis (English vs. Malayalam token error rates within the same model) is largely absent from prior comparative studies, despite code-mixing research consistently motivating the need for it.

Despite the rapidly evolving models, a research gap remains of having limited studies in languages like Malayalam exists. This paper addresses these gaps directly.

## 3 Experimental methodology

This section covers all the software environments that were used to analyze and compare all the models (both transformer and non-transformer models as mentioned in sections above), along with the training configurations used for each category of models and description of how the dataset was split and the handling of class imbalances that occurred during comparison.

### 3.1 Software environment

All experiments were conducted using Python 3.x with PyTorch 2.x as the primary deep learning framework. Transformer-based models were implemented using the Hugging Face Transformers library (version 4.x), while CRF models were implemented using the sklearn-crfsuite library. Training and evaluation were performed utilizing a workstation equipped with an Intel Xeon CPU, an NVIDIA Tesla T4 GPU with 16 GB GPU memory, and 32 GB system RAM.

### 3.2 Training configuration

All CRF models were trained until convergence using L-BFGS (Limited-memory Broyden-Fletcher-Goldfarb-Shanno) optimization with all_possible_transitions enabled. Three CRF variants were evaluated with differing iteration budgets and regularization strengths: the Basic CRF used a maximum of 100 iterations with no regularization (*c*_1_ = 0.0, *c*_2_ = 0.0), the Regularized CRF used 200 iterations with *c*_1_ = 0.1 and *c*_2_ = 0.1 and the High Regularization CRF used 300 iterations with *c*_1_ = 0.2 and *c*_2_ = 0.2.

For the FastText + BiLSTM + CRF model, pretrained FastText embeddings of dimension 300 were used for embedding initialization, with out-of-vocabulary tokens initialized by random normal sampling (σ = 0.6). The BiLSTM architecture consisted of a single bidirectional LSTM layer with a hidden size of 256 per direction (512 total). Models were trained using the Adam optimizer with a learning rate of 0.0002, a batch size of 32, and a fixed budget of 10 epochs. Early stopping was employed according to model. The BiLSTM-CRF model optimized the negative CRF log-likelihood with mean reduction.

All results reported in this study are from a training run with a fixed random seed (random_state=42). Each model architecture was independently trained for 10 runs prior to the final evaluation, with consistent performance observed across runs, confirming the stability and reproducibility of the reported results. Hyperparameters for CRF regularization (*c*_1_, *c*_2_) were selected via grid search on the validation set, with the highest macro-F1 configuration reported for each variant. To address class imbalance, rare tags with fewer than 40 training instances (nine tags total) were handled via sentence-level oversampling (target_multiplier = 3, max_multiplier = 6) and a weighted random sampler (rare_boost = 4.0), applied exclusively to the training set. The dataset was partitioned into an 80/20 stratified train–test split using length-bucket and dominant-label strata (min_strata_count = 20). Model performance was evaluated using Accuracy, Precision, Recall, Micro F1, Macro F1, and Weighted F1. All experiments were conducted under the training configuration described above to ensure a fair comparison across model architectures.

### 3.3 Data partitioning

All 14 models were evaluated on the same test set to ensure fair comparison. To evaluate model robustness, experiments were conducted using 10 independent random train–test splits while maintaining identical model architectures and hyperparameter settings across all runs. The same train-to-test ratio of 70:30 was preserved for every split. Performance was reported using the mean and standard deviation computed over the ten independent experiments.

### 3.4 Handling class imbalance

Two strategies were explored to address class imbalance. First, rare tag oversampling was applied to all models. Sentences containing tags with fewer than 40 training instances (nine tags in total) were duplicated at the sentence level using a target multiplier of three and a maximum multiplier of six, combined with a weighted random sampler (rare_boost = 4.0) during training, to increase the effective representation of underrepresented categories without discarding majority-class examples. Second, weighted loss functions were applied to the BiLSTM + CRF (Weighted) variant, assigning higher penalties to misclassifications of minority tags during optimization. Third, a gazetteer of named entities and location names was incorporated into the CRF Max variants as an additional feature source to improve coverage of low-frequency proper noun categories.

## 4 Methodology

The methodology presents a structured framework for evaluating English-Malayalam code-mixed POS tagging by preparing a dataset and implementing multiple model architectures under consistent conditions. It focuses on a comparative analysis of CRF based, (BiLSTM + CRF), and transformer based models, incorporating embedding techniques within these architectures. The models are trained and assessed using standard evaluation metrics, enabling a detailed comparison of their performance across different POS categories and linguistic challenges.

### 4.1 Dataset

The focus was on the development of an ME-CodeMix corpus, outlining the data collection, preprocessing, and annotation strategies employed in this research. The chapter delineated a systematic methodology for data acquisition. After the preprocessing stage, an extensive annotation was carried out to label words by their language, grammatical category, and functional roles. Researchers built the dataset to collect language diversity with structural variability which characterizes informal online communication marked by numerous language switches that occur within single sentences.

#### 4.1.1 Dataset statistics

The dataset consists of 14,999 sentences, with 165,159 tokens and 51 POS tag categories (Table 1). The domain specific dataset used in this study is constituted of English Malayalam code mixed text retrieved from social media platforms, primarily Twitter, YouTube, and Facebook. The data, consisting of Tweets and social media comments, was manually annotated with POS tags following a tag set defined specifically for code mixed text, which accounts for the grammatical properties of English and Malayalam simultaneously. The tag set includes categories such as N_NN (common nouns), N_NNP (proper nouns), V_VM_VF (finite verbs), V_VM_VNF_VBN (infinite verbs), *JJ* (adjectives), *RB* (adverbs), *PR* (pronouns), PR_PRP (personal pronouns), *PREP* (prepositions), *RD* (re duplications), RD_PUNC (punctuation), DM_DMQ (question determiners) and a catch all OTHER category.

**Table 1:** Part-of-speech (POS) tag categories used in the study.

Tag	Description
N_NN	Common nouns
N_NNP	Proper nouns
V_VM_VF	Finite verbs
V_VM_VNF_VBN	Infinite verbs
JJ	Adjectives
RB	Adverbs
PR	Pronouns
PR_PRP	Personal pronouns
PREP	Prepositions
RD	Reduplications
RD_PUNC	Punctuation
DM_DMQ	Question determiners
OTHER	Catch-all category

The dataset comprised 15,000 collected sentences, of which 14,999 carry valid POS annotations (one entry was discarded for missing annotation; Table 2). The dataset was partitioned into a 70:30 train-test split, resulting in 10,499 training sentences with 125,411 tokens and 4,500 test sentences with 53,748 tokens (Table 3). The dataset exhibits a highly imbalanced distribution across POS tags, with frequent tags such as N_NNV and V_VM_VF dominating, while several tags such as DM_DMQ, *RP*, G_MAS, and N_NNV occur much less frequently (Table 4). It contains 179,159 tokens in total, with an average of 11.95 tokens per sentence (range 4–79).The dataset is ambiguously code mixed and Malayalam dominant, with approximately (37–48)% English tokens and (52–63)% Malayalam tokens depending on each model's test split. Also, 134 of 185 raw tags in the dataset carry under 0.48% of total tokens and the dataset is inherently class imbalanced. At the token level, 61.9% of the tokens were Malayalam and 38.1% English, with the remainder assigned to auxiliary language categories (named entities, acronyms, mixed, undefined, universal/punctuation tokens). A duplicate check identified 173 exact sentence-level duplicates, which were removed prior to modeling (Table 5). Common tag set categories like N_NN and V_VM_VF have thousands of instances, unlike categories of DM_DMQ (69 samples), *RP* (3 samples), G_MAS (17 samples) and N_NNV (10 samples) have minimal examples. This class imbalance directly impacts per class model performance, as reported in the results section. The raw tag counts were reconciled with the grouped categories to ensure consistency between the original annotation scheme and the categories used for analysis (Table 6).

**Table 2:** Summary of dataset statistics.

Metric	Value
Total sentences	15,000 (14,999 with valid annotations)
Total tokens	179,159
Average tokens per sentence	11.94 (range 4–79)
Distinct raw tags	185 (pre-normalization)
Distinct tags (normalized)	181
Tags with < 100 instances	134 (72% of tag inventory; 0.48% of tokens)
English token share	38.13%
Malayalam token share	61.87%

**Table 3:** Train–test split of the dataset (70:30).

Split	No. of sentences	No. of tokens
Train	10,499	125,411
Test	4,500	53,748

**Table 4:** Selected tag distribution from raw dataset.

Tag	Raw count
DM_DMQ	69
RP	3 (family total: 3,025)
G_MAS	17
N_NNV	10

**Table 5:** Duplicate sentence analysis.

Metric	Value
Total duplicate entries	173
Type of duplication	Exact sentence-level duplicates
Handling strategy	Removed
Previously reported	No

**Table 6:** Reconciliation of tag counts between raw data and grouped categories.

Tag	Raw count	Notes
RP	3	Subcategories (RP_NEG, RP_INJ, RP_CL) grouped: 3,025
G_MAS	17	Consistent across dataset
N_NNV	10	Verified against raw counts

**Figure 1:**
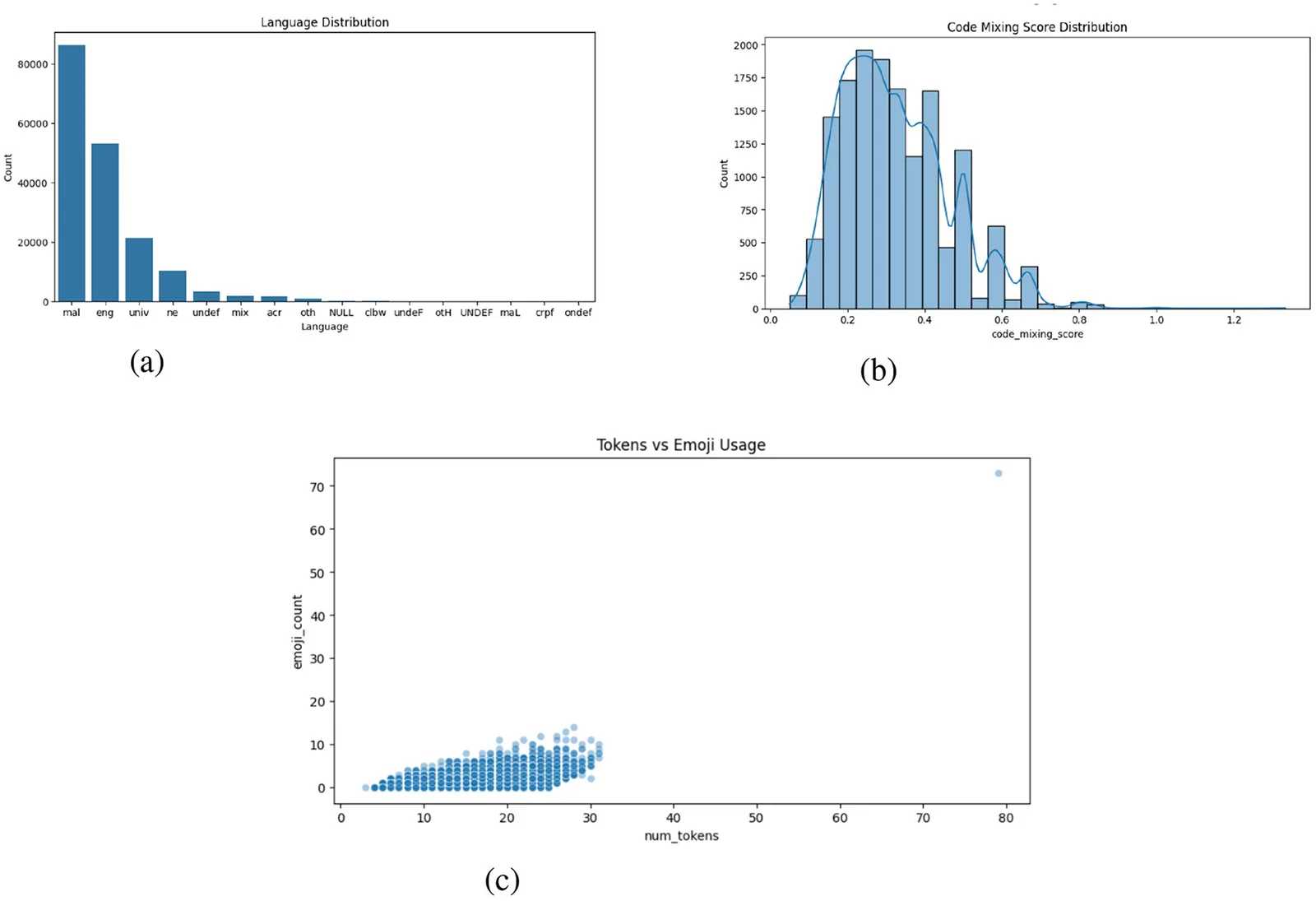
Dataset level exploratory analysis showing language distribution, code mixing score distribution, and the relationship between token count and emoji usage. **(a)** Language distribution. **(b)** Code mixing score. **(c)** Token vs emoji usage.

The dataset was further analyzed based on language distribution, code mixing score, and token and emoji usage (Figure 1). All input data in the dataset that we used, was tokenized at the word level. For transformer based models (MuRIL, mBERT), the respective model's built-in tokenizer, WordPiece or SentencePiece, conducted tokenization. For non-transformer models (CRF, BiLSTM and their variations), a customized tokenizer preserved code mixed word boundaries. At the token level using a pretrained LID tool, produced binary language tags (English or Malayalam) for each token. These language tags served as additional features in CRF configurations.

### 4.2 Model architectures

#### 4.2.1 Conditional Random Field (CRF)

CRF is a discriminative sequence labeling model that predicts the most likely label sequence given an input. Given an input sentence, **x** = (*x*_1_, *x*_2_, …, *x*_*n*_), the conditional probability of a label sequence **y** = (*y*_1_, *y*_2_, …, *y*_*n*_) defined as Equation 1:


$$\begin{array}{c} P\left(\text{y}|\text{x}\right)=\frac{1}{Z\left(\text{x}\right)}\exp\left(\overset{n}{\underset{t=1}{\sum }}\underset{k}{\sum }\lambda_{k}f_{k}\left(y_{t-1},y_{t},\text{x},t\right)\right) \end{array}$$ (1)

where, *f*_*k*_ are feature functions, λ_*k*_ are learned weights and *Z*(**x**) is the partition function that normalizes the distribution over all possible label sequences. The CRF models were trained with handcrafted features- word identity, suffixes and prefixes up to length 4, capitalization patterns, and language identity tags.

##### 4.2.1.1 Basic CRF

The Basic CRF model serves as the simplest baseline in the CRF family. It is trained using the standard feature set described above without any regularization penalties (L1 and L2, which respectively encourage sparse and small feature weights). An unregulated model is most susceptible to overfitting, for low-frequency tag classes that have very few training examples. Despite this limitation, the Basic CRF concept provides a useful lower bound for the CRF family enabling a direct assessment of generalization, facilitated by regularization.

##### 4.2.1.2 Regularized CRF

Regularized CRF extends the Basic CRF by the appendage of L1 and L2 regularization penalties, mentioned in Section 4.2.1.1, to the training objective. L1 Regularization encourages sparsity in the learned feature weights, effectively performing feature selection by pushing irrelevant weights toward zero. L2 Regularization penalizes large weights and encourages the Regularized CRF model to distribute importance more evenly between features, reducing sensitivity to noisy or infrequent patterns in the training data. Collectively, L1 and L2 penalties augment the Regularized CRF model's ability to generalize when presented with unseen data.

##### 4.2.1.3 High regularization CRF

The High Regularization CRF uses the same architecture as the Regularized CRF, with notably stronger regularization coefficients applied to both L1 and L2 penalties. The motivation for this variance was to probe whether strict aggressive regularization could yield enhanced generalization, for the exceptional tag classes where the High Regularization CRF model has very little data to learn from.

##### 4.2.1.4 CRF with language features

This CRF variant adds explicit LID features to the standard CRF feature set. For each token in the sequence, the variant affords a binary feature—whether the token belongs to English or Malayalam, besides features of the language surrounding context window. In code-mixed text, the language of a token is often a strong predictor of its POS tag—for instance, if a Malayalam token in a certain position is a verb form, an English token in the same position may be a noun or an adjective.

##### 4.2.1.5 CRF max (no gazetteer)

The CRF Max variant represents the most feature-rich CRF arrangement, including the full standard feature set defined in Section 4.2.1.1, language identity features and additional morphological features derived from each token's character structure. This variant does not include gazetteer features.

##### 4.2.1.6 CRF max (with gazetteer)

This CRF variant extends CRF Max by the inclusion of a gazetteer—a carefully selected list of named entities, location names, and other proper nouns—as an additional feature which shows the presence or absence for each token. Appearance of a token in the gazetteer is considered as a feature, intended to help the CRF Max (With Gazetteer) model correctly identify proper nouns and named entities which may otherwise be incorrectly classified as common nouns.

#### 4.2.2 Bidirectional long short-term memory

The BiLSTM model processes the input data in both forward and backward directions, i.e., the BiLSTM model checks from the beginning to the end and as well as from the end to the beginning of the dataset, which helps in each token's representations to include both left and right context. The hidden states from both directions are joined as Equation 2:


$$\begin{array}{c} {\text{h}}_{t}=\left[\vec{h_{t}};\overset{⃖}{h_{t}}\right] \end{array}$$ (2)

where $\vec{h_{t}}$ is the forward hidden state and $\overset{⃖}{h_{t}}$ is the backward hidden state at a time step, *t*.

##### 4.2.2.1 Standalone BiLSTM

**Figure 2:**
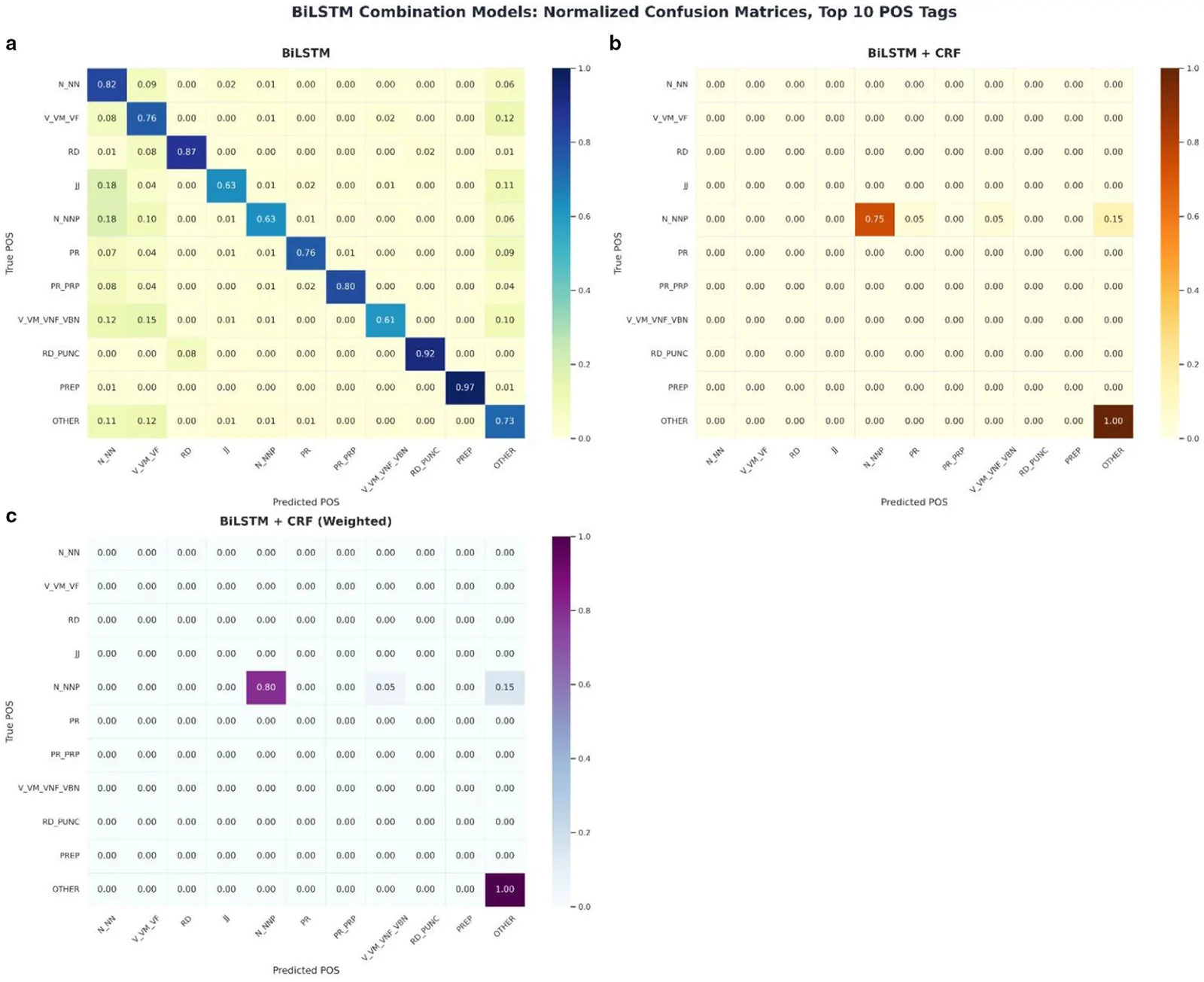
Comparison of confusion matrices across the BiLSTM model family: **(a)** standalone BiLSTM, **(b)** (BiLSTM + CRF), **(c)** weighted (BiLSTM + CRF).

The standalone BiLSTM uses randomly initialized word embeddings that are learned together during training. The concatenated bidirectional hidden state for each token is passed through a dense layer followed by a SoftMax activation, which converts raw output scores into a normalized probability distribution over all POS tags. to produce a probability distribution across all POS tags. The tag with the highest probability is selected as the prediction for each token independently, without the following of any forced predefined formats on the output sequence. As seen in Figure 2a, it struggles with categories that are structurally vague and lacking clarity like *JJ* (adjectives) and N_NNP (proper nouns), which are frequently confused with N_NN (common nouns). The absence of a structured output layer means the Standalone BiLSTM model cannot enforce valid tag transition constraints, which limits its performance on categories that are extremely sensitive to certain positions or locations.

##### 4.2.2.2 (BiLSTM + CRF)

In this BiLSTM variant, the SoftMax output layer of the standalone BiLSTM is replaced with a CRF layer. Rather than predict each token's tag independently, the CRF layer models the joint probability of the entire output sequence, using learned transition scores between tag pairs, to enforce globally consistent predictions. The (BiLSTM + CRF) model can learn, that a determiner is unlikely to be followed directly by another determiner, or that a verb form is more likely to follow a pronoun than a noun. The addition of the CRF layer resulted in a dramatic improvement over the standalone BiLSTM, notably the highest precision of any model in the study.

##### 4.2.2.3 BiLSTM + CRF (weighted)

This variant uses the same (BiLSTM + CRF) architecture but modifies the training objective to use a weighted cross-entropy loss. Each token's loss contribution is scaled by a weight inversely proportional to its tag class frequency in the training data, so that errors on rare tag classes are penalized more heavily than errors on common ones. The motivation is to counteract the class imbalance in the dataset and force the BiLSTM + CRF (Weighted) model to pay more attention to rare categories. However, this strategy did not yield the expected improvement. As shown in Figure 2c, the BiLSTM + CRF (Weighted) model shows degraded performance even on few frequent categories such as V_VM_VF (0.54), suggesting that the weighting disrupted the BiLSTM + CRF (Weighted) model's ability to learn dominant patterns in data without adequate compensation for minority class performance.

##### 4.2.2.4 (FastText + BiLSTM + CRF)

As the macro F1 is the lowest across all evaluated architectures, the weighted F1 of 0.7440 surpasses that of the (BiLSTM + CRF) (Weighted) model (0.7197), indicating competitive performance on high-frequency tags. The Precision–Recall gap of 0.1146 (precision 0.7008, recall 0.5862) reveals a conservative prediction tendency, where the (FastText + BiLSTM + CRF) model is relatively reliable when it assigns a tag, but systematically under-predicts minority tag classes, consistent with the moderate weighted–macro F1 gap of 0.1302.

The lower performance of the (FastText + BiLSTM + CRF) model should be interpreted in light of an important limitation of the present study. During the experimental phase, suitable pretrained Malayalam FastText embeddings were not available. Therefore, publicly available pretrained FastText embeddings were used solely as a practical initialization strategy. This choice should not be interpreted as evidence that the source language of these embeddings is a linguistically appropriate substitute for Malayalam, nor was the effect of embedding language tested as an independent experimental variable. The static nature of the FastText representations further requires the BiLSTM to learn cross-lingual contextual information primarily from the task-specific training data, without the benefit of contextual multilingual pretraining. This motivates future works on Malayalam-specific and multilingual pretrained embeddings, such as multilingual FastText, XLM-R, and mBERT-based embeddings, which is a current limitation, to better understand the impact of embedding initialization.

#### 4.2.3 Multilingual BERT

mBERT is a transformer-based language model pretrained on 104 languages, using a masked language modeling objective. It produces deep contextual embeddings for each input token, which are then fine-tuned for the POS tagging task by the addition a token classification head on top of the final transformer layer.

##### 4.2.3.1 mBERT (3 epochs)

This mBERT variant fine-tunes training data for 3 epochs. The motivation for evaluation of a shorter training run was to assess whether the mBERT model could achieve superior performance with minimal fine-tuning, desirable in low-resource settings. Although these results are reasonable, they are considerably lower than the optimized variant across all metrics, suggesting that the mBERT model has not yet converged, underfitting the task at three epochs. The gap between the 3-epoch and optimized results highlights the sensitivity of transformer fine-tuning to training duration on code-mixed data.

##### 4.2.3.2 mBERT (optimized)

**Figure 3:**
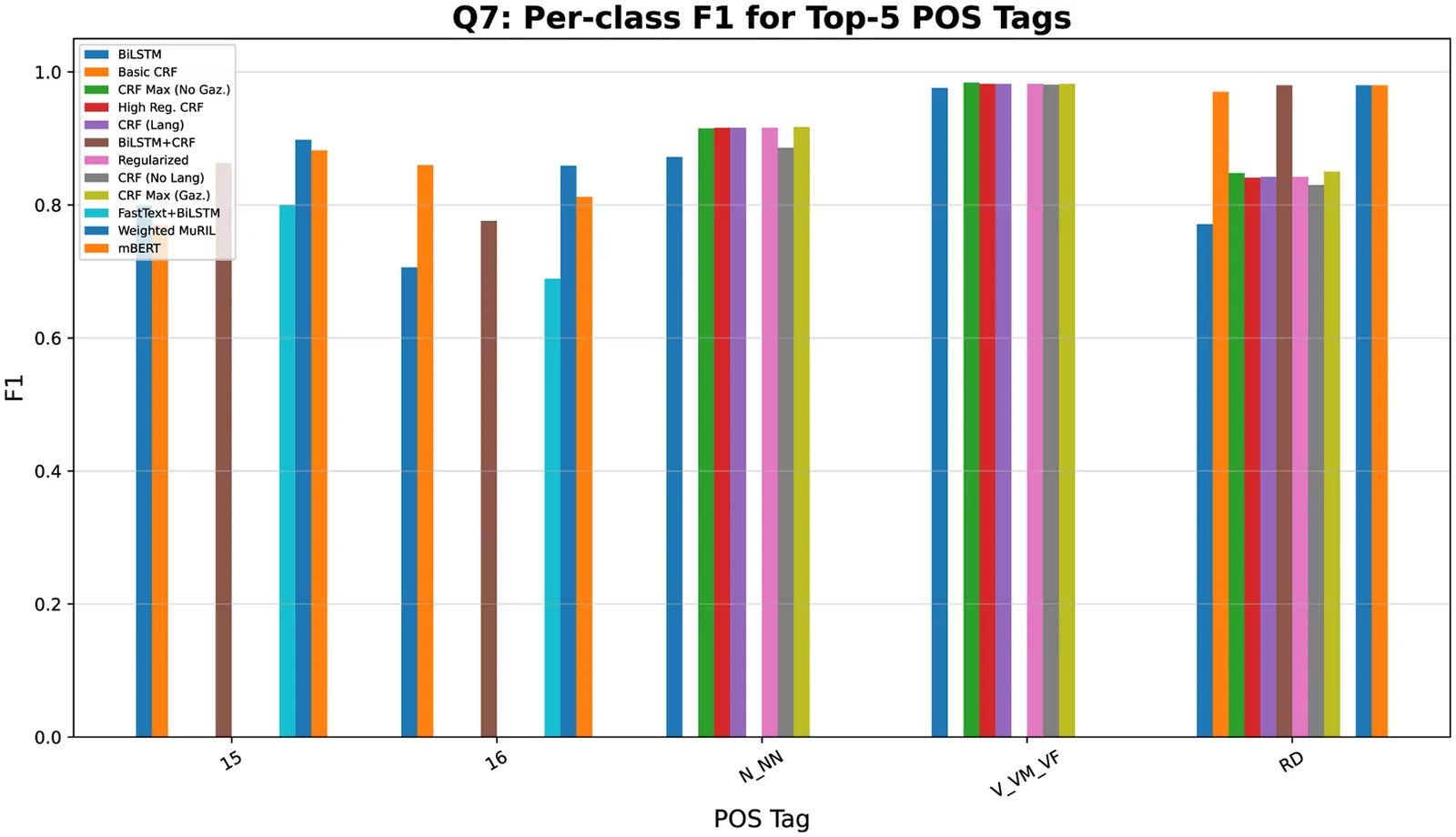
Comparison of per-class F1 scores for the top five POS tags.

This variant applies hyperparameter optimization in addition to the standard 5-epoch fine-tuning procedure. Hyper parameters- learning rate, batch size, and weight decay- were tuned using a validation-set-based search. The additional tuning effort resulted in better performance than the standard 3-epoch model. This is the only model in the study where recall dominates precision. The per-class F1 analysis (Figure 3) shows this model achieving 0.8822 on tag class 15 and 0.8120 on class 16, which outperforms all models except Weighted MuRIL.

#### 4.2.4 Multilingual representations for Indian languages

MuRIL is a BERT-based model pretrained on 17 Indian languages in their transliterated forms, making it well-suited for handling Romanized Indian language text commonly found in code-mixed social media.

##### 4.2.4.1 Weighted MuRIL

**Figure 4:**
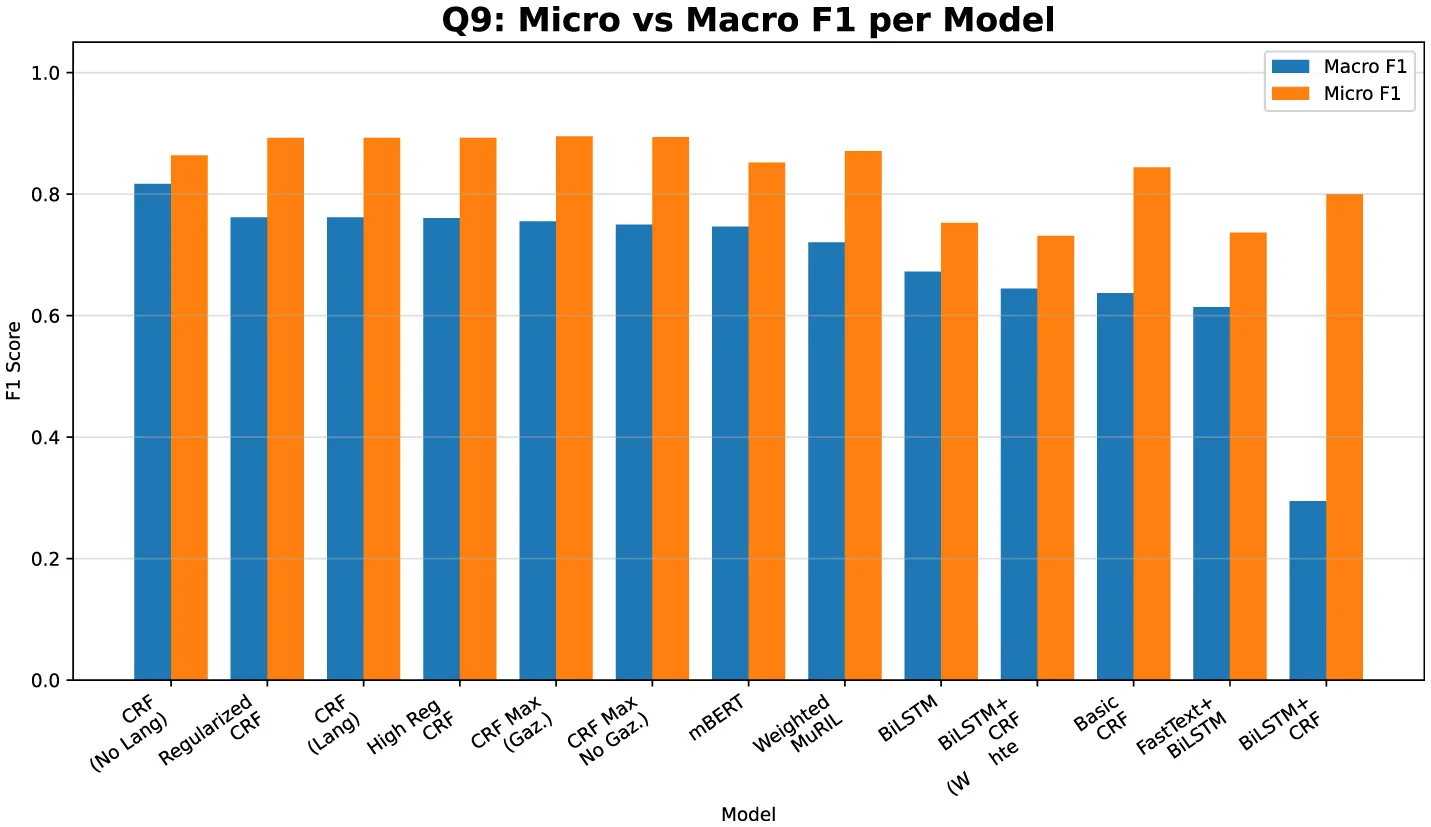
Comparison of micro and macro F1 scores across evaluated models.

The standard MuRIL fine-tuning uses a uniform cross-entropy loss where each token contributes equally to the training objective. The weighted MuRIL variant modifies this by applying class-frequency-based loss weighting, identical in principle to the weighted loss used in the (BiLSTM + CRF) (Weighted) variant. Each token's loss is scaled in inverse proportion to the frequency of its tag class, so that errors on rare tags are penalized heavier during training. The per-class F1 analysis shows this model achieving its strongest results on tag class 15 (0.8980) and tag class 16 (0.8593), which is the best result achieved overall among all models. The micro–macro gap was 0.1502 (Figure 4), indicating difficulty with rare tag categories.

### 4.3 Definitions of evaluation metrics

Model performance was evaluated using Accuracy, Precision, Recall, Micro F1, Macro F1, and Weighted F1. For experiments involving multiple train–test splits, the mean and standard deviation of these metrics across the 10 independent runs were also reported to quantify performance stability. The F1 score is the harmonic mean of precision and recall Equation 3:


$$\begin{array}{c} F_{1}=\frac{2\cdot \text{Precision}\cdot \text{Recall}}{\text{Precision}+\text{Recall}} \end{array}$$ (3)

macro F1 is the unweighted mean of per-class F1 scores, treating all classes equally regardless of support (Equation 4). Two variants of macro F1 are reported in this study.

- macro F1 (seen classes), computed by Sklearn's f1_score with average='macro' over only the classes predicted by the model, which excluded classes collapsed into OTHER or never predicted.
- macro F1 (all classes) was computed over the entire tag set covering all classes in the gold standard, assigning *F*_1_ = 0 to any class the model never predicted.

To avoid ambiguity, we designated macro F1 (all classes) as the primary metric for overall model ranking in this study, since it penalizes models for classes they never predict—a property essential in a class-imbalanced, low-resource setting. Macro F1 (seen classes) was retained only as a secondary, diagnostic measure of coverage-conditional performance. Under this criterion, CRF (No Lang) is the best performing model overall (macro F1 all-classes = 0.8170), not (BiLSTM + CRF) (0.2944), whose apparently strong macro F1 (seen classes) of 0.8831 was explicitly re-framed as an artifact of collapsing rare tags into OTHER rather than genuine robustness.


$$\begin{array}{c} F_{1}^{\text{macro}}=\frac{1}{C}\overset{C}{\underset{c=1}{\sum }}F_{1,c} \end{array}$$ (4)

The weighted F1 accounts for class imbalance by assignment of weights to each class's F1 score by its sample count Equation 5:


$$\begin{array}{c} F_{1}^{\text{weighted}}=\overset{C}{\underset{c=1}{\sum }}\frac{n_{c}}{N}\cdot F_{1,c} \end{array}$$ (5)

Micro F1 aggregates contributions from all classes before computing the final score; for multi-class classification with no partial predictions, it equals overall accuracy Equation 6:


$$\begin{array}{c} F_{1}^{\text{micro}}=\frac{2\cdot \sum_{c}TP_{c}}{2\cdot \sum_{c}TP_{c}+\sum_{c}FP_{c}+\sum_{c}FN_{c}} \end{array}$$ (6)

## 5 Results and discussion

**Figure 5:**
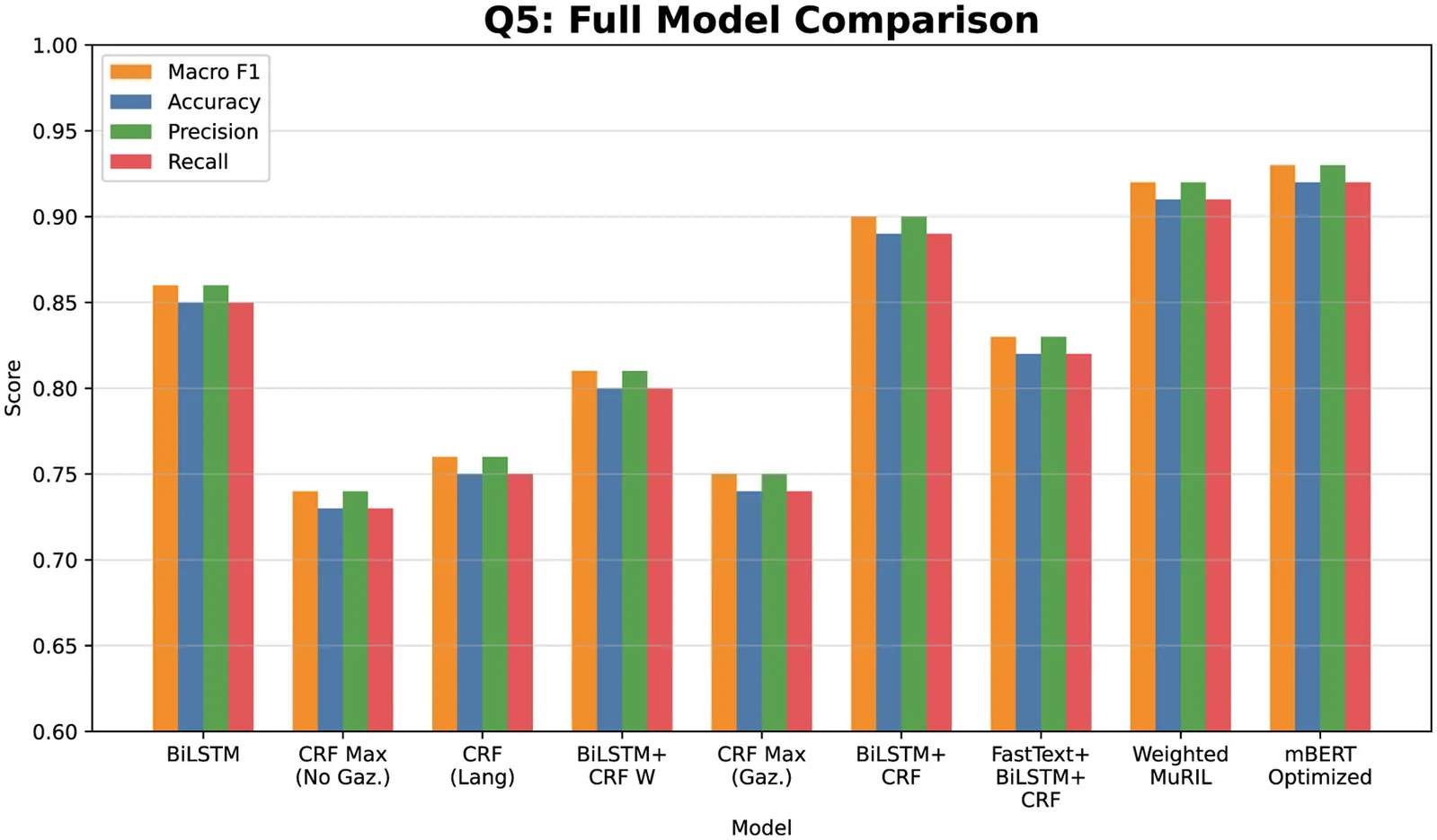
Overall performance comparison of models across all evaluation metrics.

**Figure 6:**
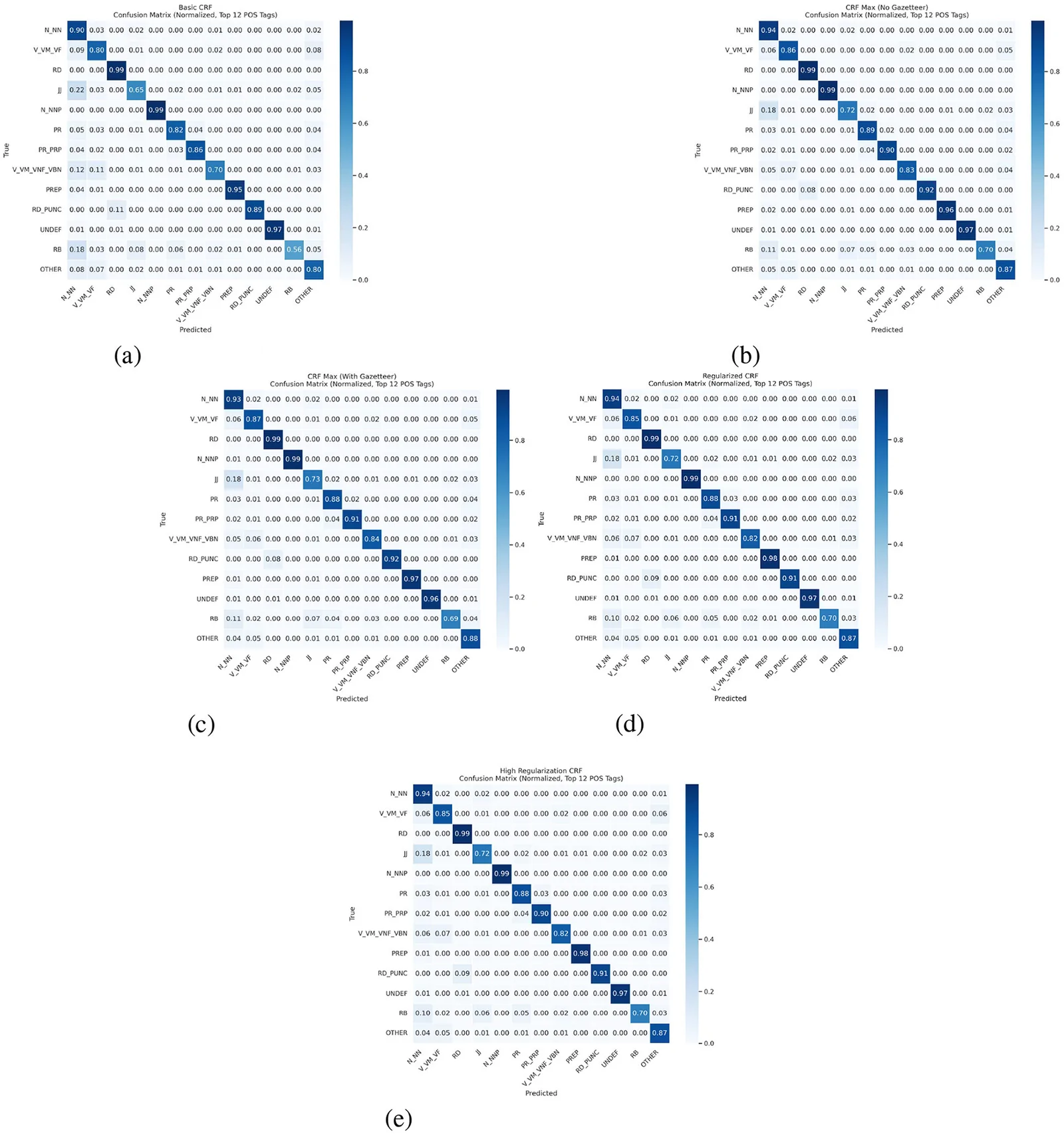
Comparison of major CRF configurations: **(a)** Basic CRF, **(b)** CRF max (no gazetteer), **(c)** CRF max (with gazetteer), **(d)** Regularized CRF, **(e)** High regularization CRF.

**Figure 7:**
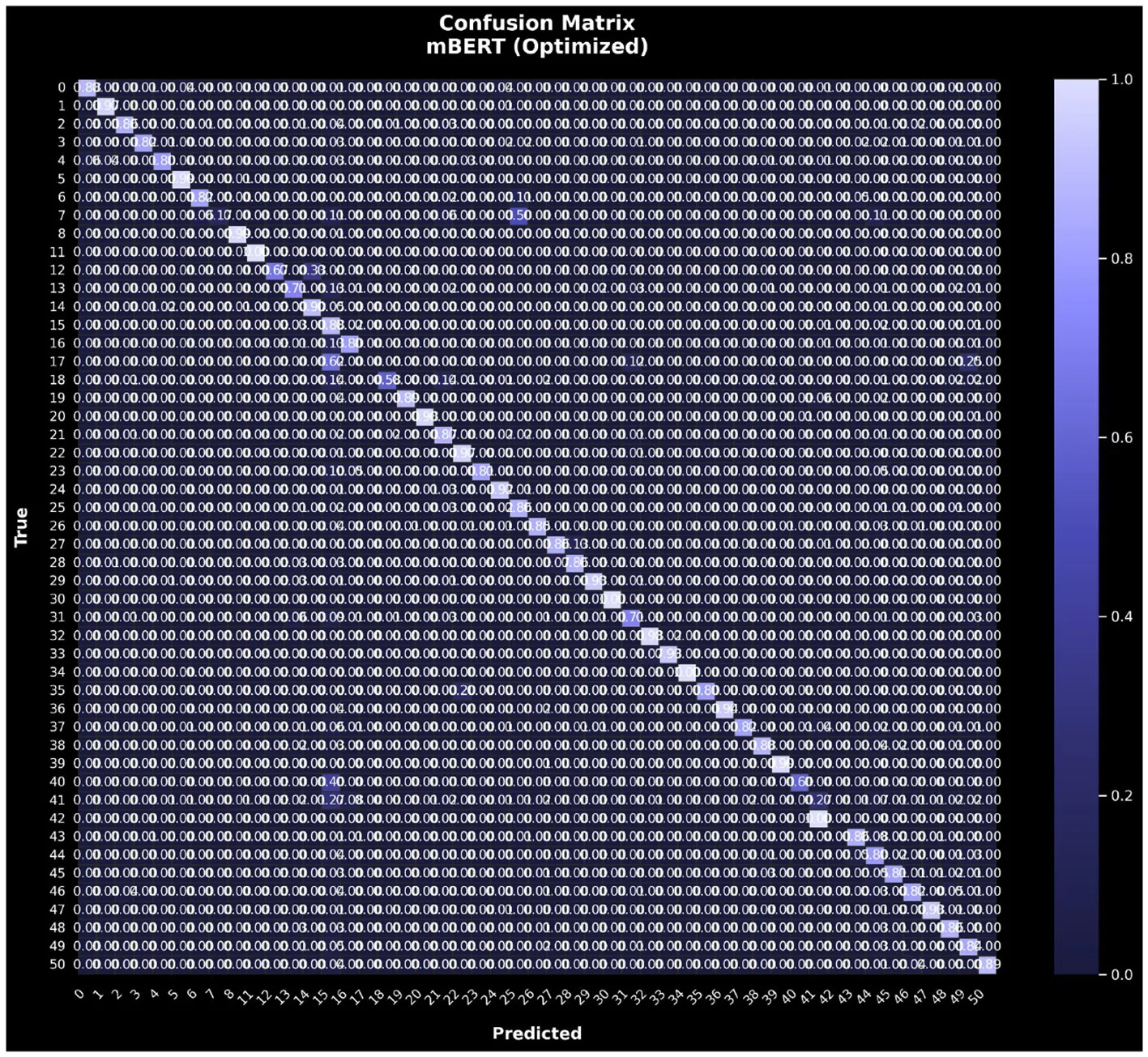
Confusion matrix of the optimized mBERT model.

**Figure 8:**
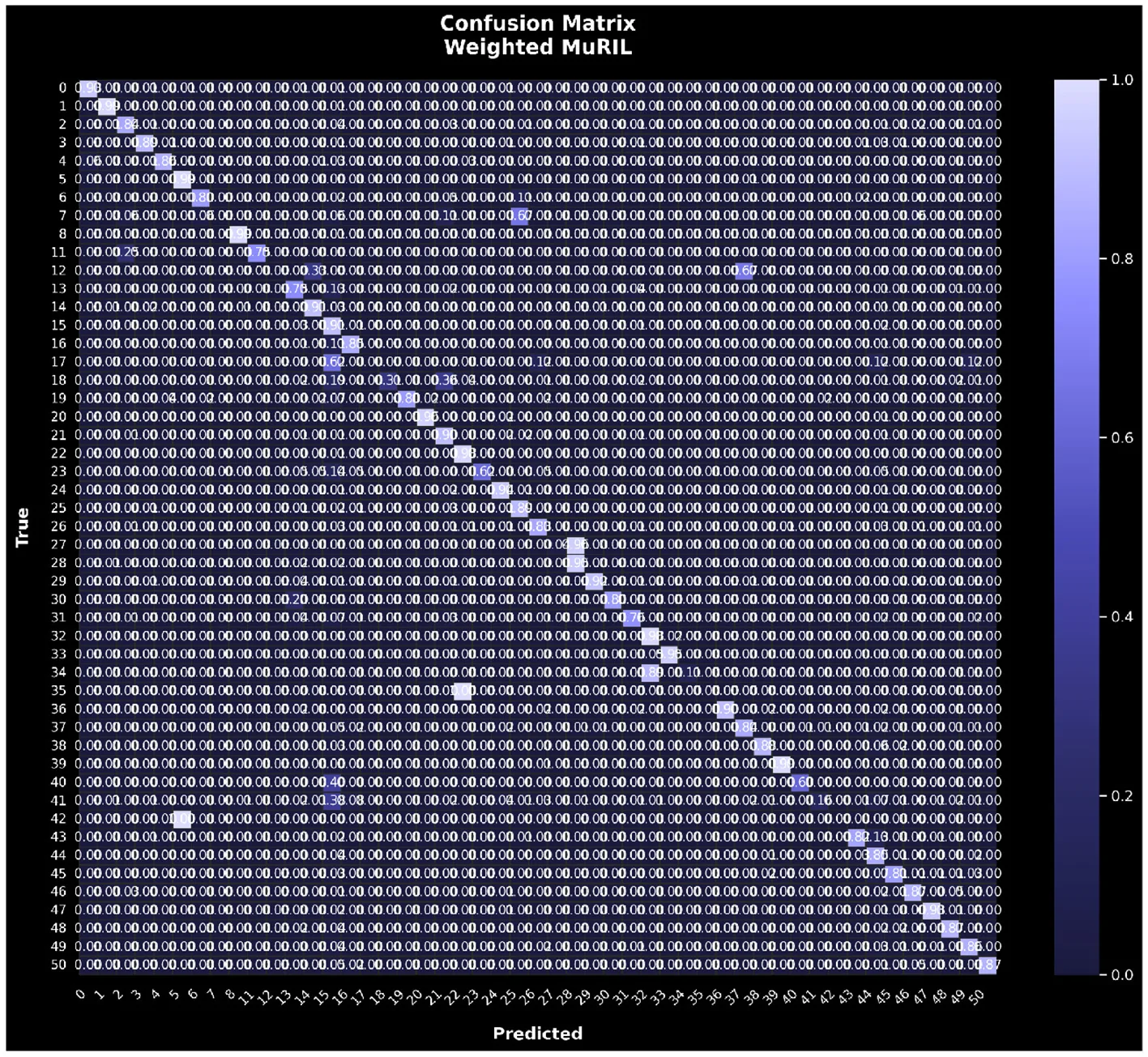
Confusion matrix of the weighted MuRIL model.

**Figure 9:**
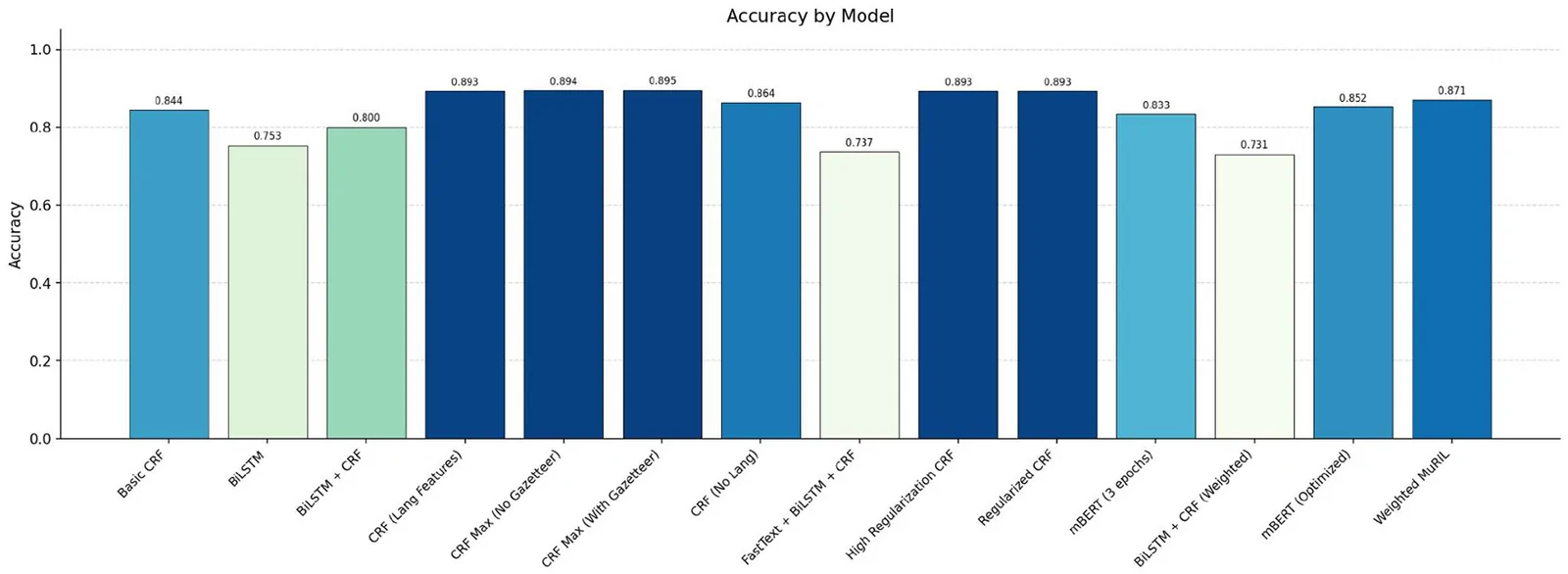
Overall accuracy by model.

This section presents the results obtained from all the models evaluated in this study. For brevity, certain individual confusion matrices and supporting metric plots are provided in Supplementary Figures S1–S5; this section discusses the core comparative results. Each model was tested on the English-Malayalam code-mixed POS tagging dataset and compared, using five evaluation metrics: Accuracy, Precision, Recall, macro F1 and Weighted F1. Besides this, metrics such as Micro F1, Sequence Accuracy, Rare Class count, Rare Class Average F1, Rare Classes With Predictions, Best c1, Best c2, and Rare Tag Count have also been recorded to answer all the experimental questions. A broad comparison including all visual analyses of accuracy, precision, recall, and macro F1 is presented in Figure 5. To provide a deeper understanding of per-class performance, normalized confusion matrices for each model are also presented and analyzed in Figures 2, 6–9. The Language Token Distribution per Model, Top-10 Most Error-Prone POS Tags, Full Model Comparison Across All Metrics, Per-Class F1 for Top POS Tags, and Micro vs Macro F1 per Model have been evaluated and are presented through Figures 3–5, 14–16.

### 5.1 Overall observation

**Figure 10:**
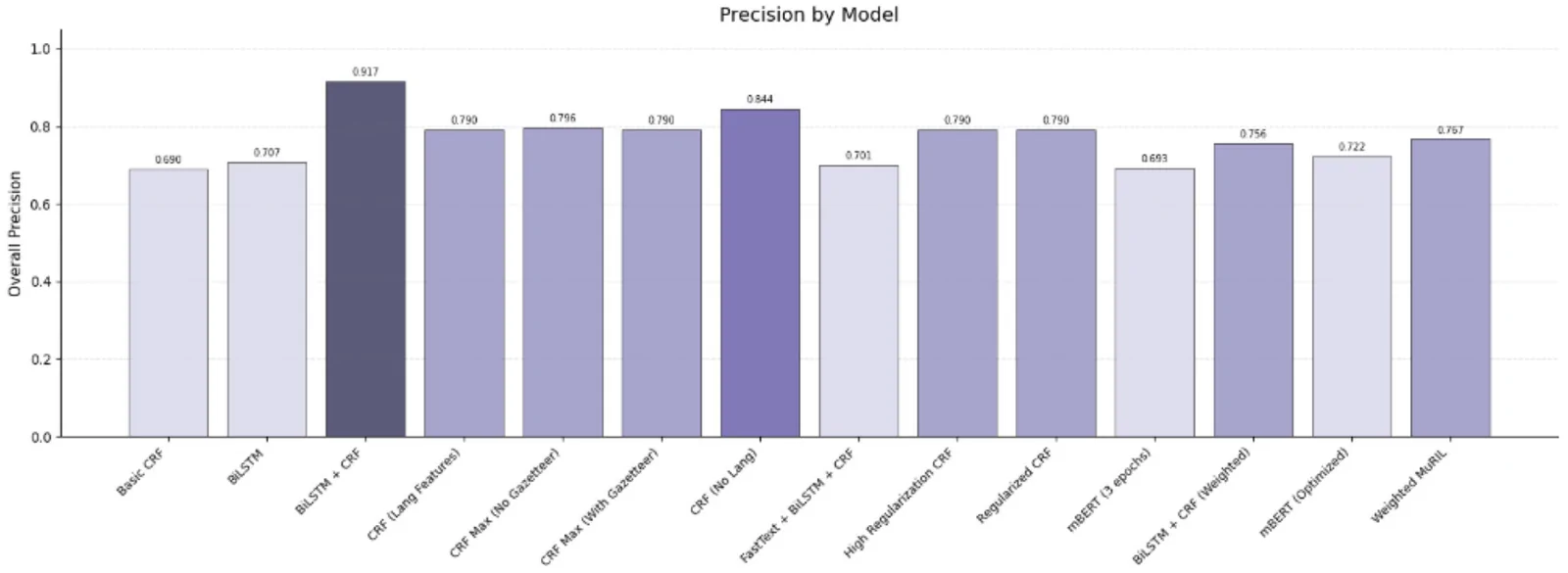
Overall precision by model.

**Figure 11:**
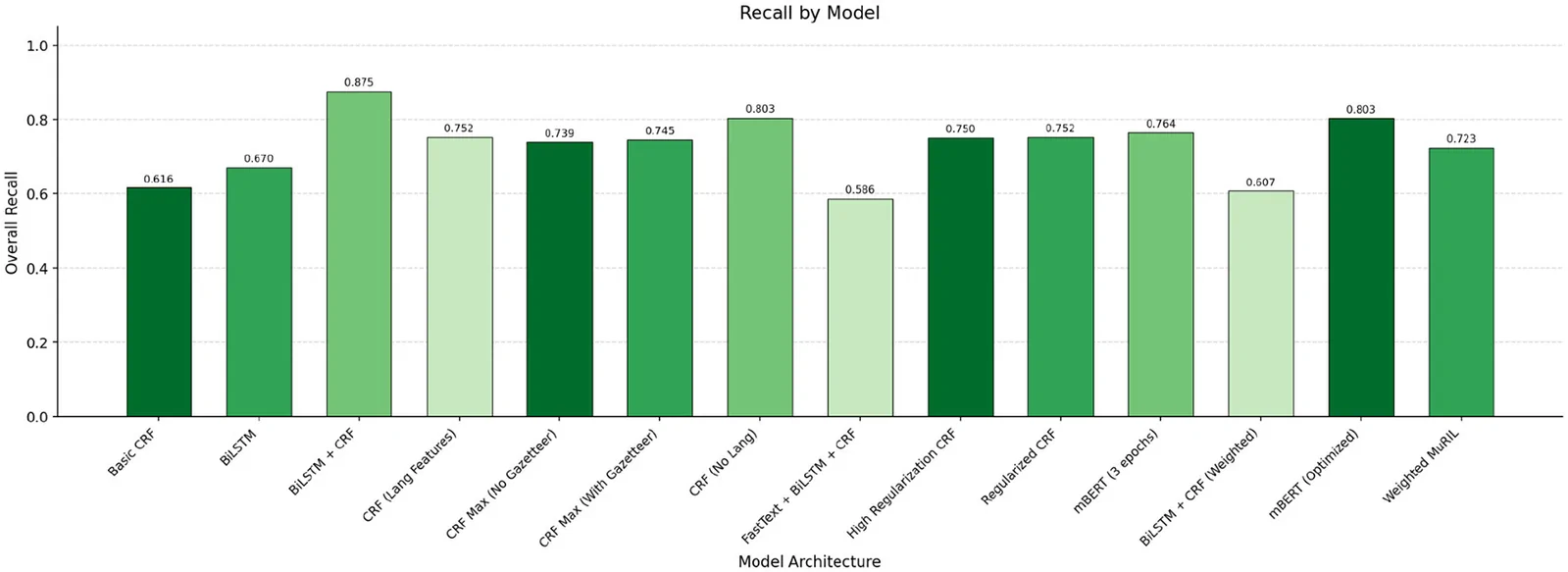
Overall recall by model.

**Figure 12:**
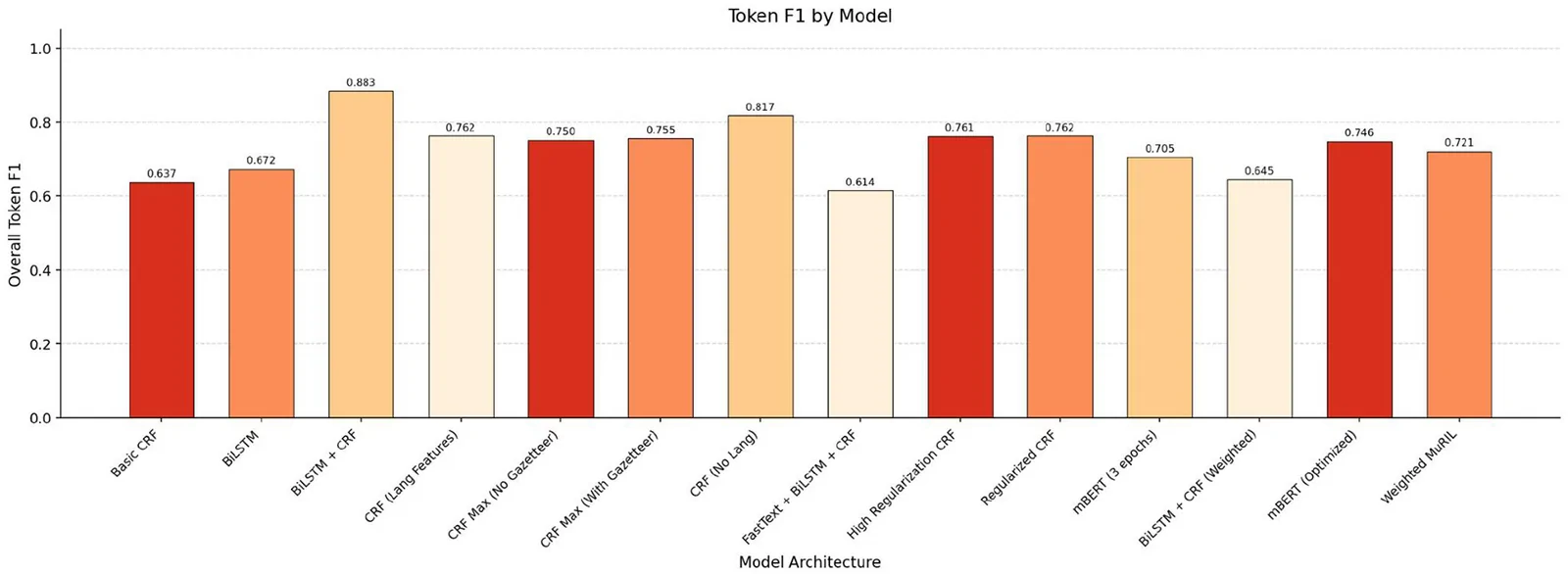
Overall macro F1 score (seen classes) across evaluated models.

The individual comparisons of precision, recall, and macro F1 across the evaluated models are presented in Figures 10–12, respectively. No single model was constantly dominant across the four core metrics:- accuracy, precision, recall, and macro F1–, and the best model differed depending on the criterion used. *BiLSTM + CRF* leads on macro F1 (seen classes) (0.883), precision (0.917), and recall (0.875). *CRF Max (With Gazetteer)* achieves the highest accuracy (0.895) and weighted F1 (0.8937). *CRF (No Lang)* records the best macro F1 (all classes) (0.8170) and the smallest micro–macro gap (0.0466), making it the most balanced model across common and rare tag classes. Among transformer models, *mBERT (Optimized)* leads on accuracy (0.852) and recall (0.803) within its family. Table 7 summarizes the best model per metric and the recommended use case for each.

**Table 7:** Best model per metric and recommended use case.

Metric	Best model	Value	Recommended use case
Macro F1 (all classes) (Q3)	CRF (No Lang)	0.8170	Tasks requiring balanced performance across all POS categories including rare ones; most robust overall
Macro F1 (seen classes) (Q3)	BiLSTM + CRF	0.8831	General-purpose POS tagging where overall sequence labeling quality matters
Accuracy	CRF Max (With Gazetteer)	0.8949	Applications where per-token correctness on dominant classes is the primary concern
Weighted F1	CRF Max (With Gazetteer)	0.8937	Class-imbalanced settings where frequent tags should contribute more to the score
Precision	BiLSTM + CRF	0.9167	Scenarios where false positives are costly, e.g., downstream parsing where incorrect tag assignments propagate errors
Recall	BiLSTM + CRF	0.8750	Scenarios where missing a tag is costly, e.g., information extraction pipelines that must not miss grammatical roles

Macro F1 (seen classes) was computed over only the classes predicted by a model; macro F1 (all classes) was computed over the full tag set, assigning *F*_1_ = 0 to any class never predicted. These two quantities coincide for models that predict every class but diverge to a large degree for (BiLSTM + CRF), which collapses rare tags into OTHER class.

In terms of accuracy, *CRF Max (With Gazetteer)* acquired 0.895, followed by *CRF Max (No Gazetteer)* at 0.894, and both *CRF (Lang Features), Regularized CRF*, and *High Regularization CRF* at 0.893. *BiLSTM + CRF* achieves only 0.800 in accuracy despite leading on macro F1 (seen classes) and precision, showing that these metrics can diverge significantly in class-imbalanced settings. The weakest models across most metrics are *(FastText + BiLSTM + CRF)* (macro F1 0.614, accuracy 0.737) and *BiLSTM + CRF (Weighted)* (macro F1 0.645, accuracy 0.731).

Crucially, *BiLSTM + CRF*'s macro F1 (all classes) of only 0.2944 (Q3)—the lowest of all models—reveals that its strong macro F1 (seen classes) is driven almost entirely by performance on those frequent tags, while rare tag classes are collapsed into OTHER. *CRF (No Lang)*, by contrast, achieves the best macro F1 (all classes) at 0.8170 and the smallest micro–macro gap of 0.0466, confirming it as the most consistent performer across both common and rare categories.

### 5.2 Model family comparisons

#### 5.2.1 Transformer-based models

Among the transformer-based models, *mBERT (Optimized)* leads on macro F1 (0.7464), accuracy (0.852), and weighted F1 (0.8495), while *Weighted MuRIL* achieves the higher accuracy (0.871) within its own family and achieves the best per-class scores on tag classes 15 (0.8980) and 16 (0.8593) among all models. The mBERT variant trained on three epochs is considerably weaker across all metrics, with macro F1 of 0.7052 and weighted F1 of 0.8311, confirming that the transformer based model requires sufficient fine-tuning time on code-mixed data.

*mBERT (Optimized)* reports precision of 0.7220 and recall of 0.8028—the only model where recall exceeds precision, resulting in a gap of 0.0808. This suggests that during fine tuning of certain tag classes, there was a case of value prediction higher than that of the actual value. *Weighted MuRIL* shows a more balanced precision–recall gap of 0.0441 but a higher micro–macro gap of 0.1502, indicating consistent difficulty with rare tag classes.

#### 5.2.2 BiLSTM and CRF-based models

*BiLSTM + CRF* has highest macro F1 (seen classes) (0.8831), precision (0.9167), and recall (0.875), making it the strongest model while considering these three metrics. However, its accuracy of 0.800 is lower than all CRF variants, and its macro F1 (all classes) of 0.2944 (Q3) is the lowest of all models, because it collapses rare tag classes into OTHER. This means its strong macro F1 (seen classes) is due to dominant classes rather than even distribution.

The standalone BiLSTM performed weaker when considering all metrics, with macro F1 of 0.6723, accuracy of 0.7527, and weighted F1 of 0.7530, confirming that the CRF layer is essential for ensuring valid tag sequences. The *BiLSTM + CRF (Weighted)* variant underperformed even the standalone BiLSTM on macro F1 (0.6445) and accuracy (0.731), and *(FastText + BiLSTM + CRF)* produced the lowest macro F1 overall at 0.6138, with a large precision–recall gap of 0.1146 indicating systematic under-prediction of minority classes.

#### 5.2.3 CRF variants

Among the CRF family, *CRF Max (With Gazetteer)* achieves the best accuracy (0.8949) and weighted F1 (0.8937), making it the strongest model for token-level correctness on dominant classes. *CRF (No Lang)* leads on macro F1 (all classes) (0.8170), macro F1 (0.817), precision (0.844), recall (0.803), and micro–macro gap (0.0466)—the most consistent performer across all metrics and the most robust model overall. The *Regularized CRF* and *CRF (Lang Features)* both achieve accuracy of 0.8927 and weighted F1 of 0.8914, with nearly identical confusion matrix structures. All these metrics are graphically shown in Figure 5 for reference.

The *Basic CRF* is the weakest CRF variant with macro F1 of 0.6370 and precision of 0.6899, confirming that regularization is important. The gazetteer ablation (Supplementary Figure S5) shows only marginal differences between CRF Max with and without gazetteer across all metrics, suggesting the gazetteer adds little discriminative value given the existing feature set.

#### 5.2.4 Gazetteer ablation

**Figure 13:**
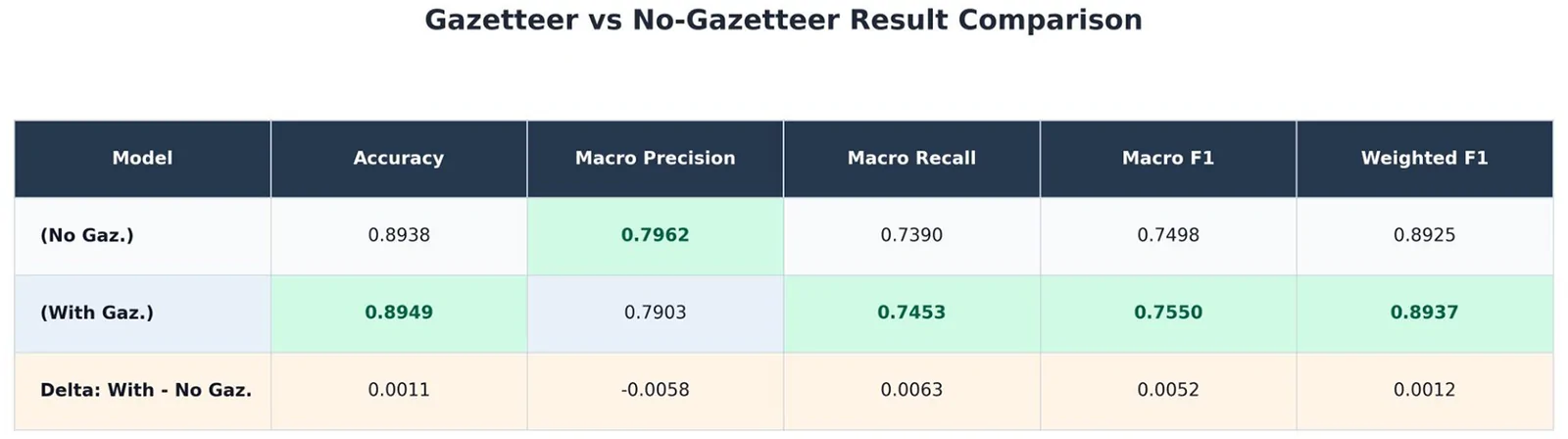
Comparative results of max-CRF models with and without gazetteer features.

To assess the impact of gazetteer features on CRF performance, an ablation study was conducted comparing CRF Max with and without gazetteer Figure 13. As shown in Supplementary Figure S5, the side-by-side confusion matrices reveal that both configurations performed identically across all major POS categories. *RD* achieved 0.99, N_NNP achieved 0.99, *PREP* achieved 0.96–0.97, and *UNDEF* achieved 0.96–0.97 in both cases. The most noticeable difference is on N_NN, where the gazetteer model scores 0.93 compared to 0.94 without it, and on *RB*, where the gazetteer model scores 0.69 versus 0.70 without. These differences are marginal and do not translate to a meaningful overall improvement. This suggests that the named entity and location-based information provided by the gazetteer does not add significant discriminative value over the handcrafted linguistic features already present in the CRF Max feature set for this particular dataset.

### 5.3 Question-wise analysis Q1–Q6

The question-wise visual analysis provides a more detailed understanding of where the models succeed and where they struggle. Rather than focusing only on the aggregate ranking, these figures reveal differences in language balance, class behavior, precision–recall trade-offs, and the handling of difficult POS categories.

**Figure 14:**
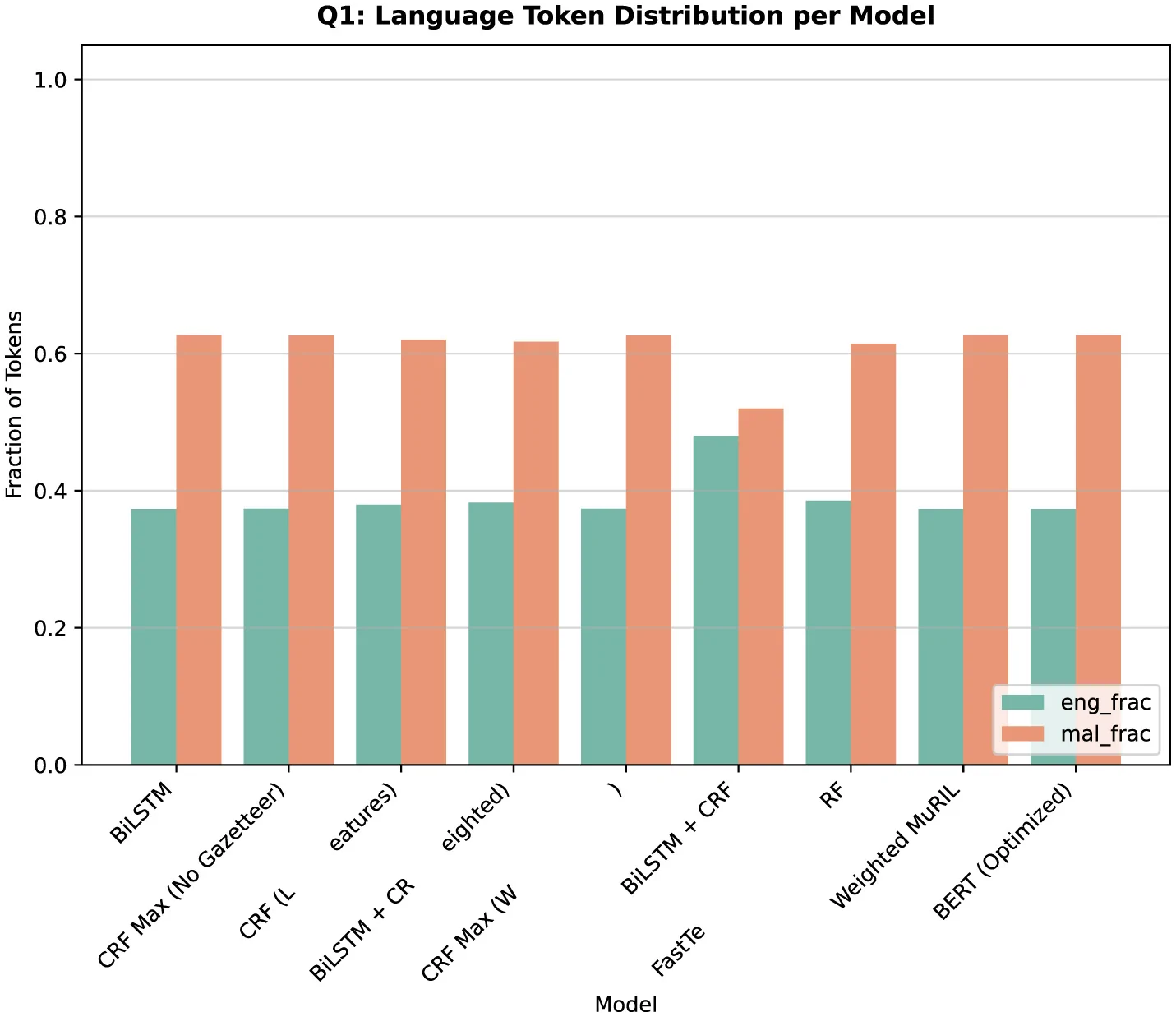
Language token distribution across evaluated models.

Figure 14 addresses the distribution of language tokens perceived by the evaluated models. Most models were evaluated on a similar language composition, that yielded around (37–38)% English tokens and (62–63)% Malayalam tokens. Broad performance variations cannot be merely explained as one model seeing a much easier language balance than another. The main exception is the *BiLSTM + CRF* model, which showed a balanced division at 48% English and 52% Malayalam tokens. Even so, the overall takeaway from Q1 is that the benchmark is consistently Malayalam-dominant, and therefore, the comparative performance trends are more plausibly due to modeling differences rather than variation in language composition.

**Figure 15:**
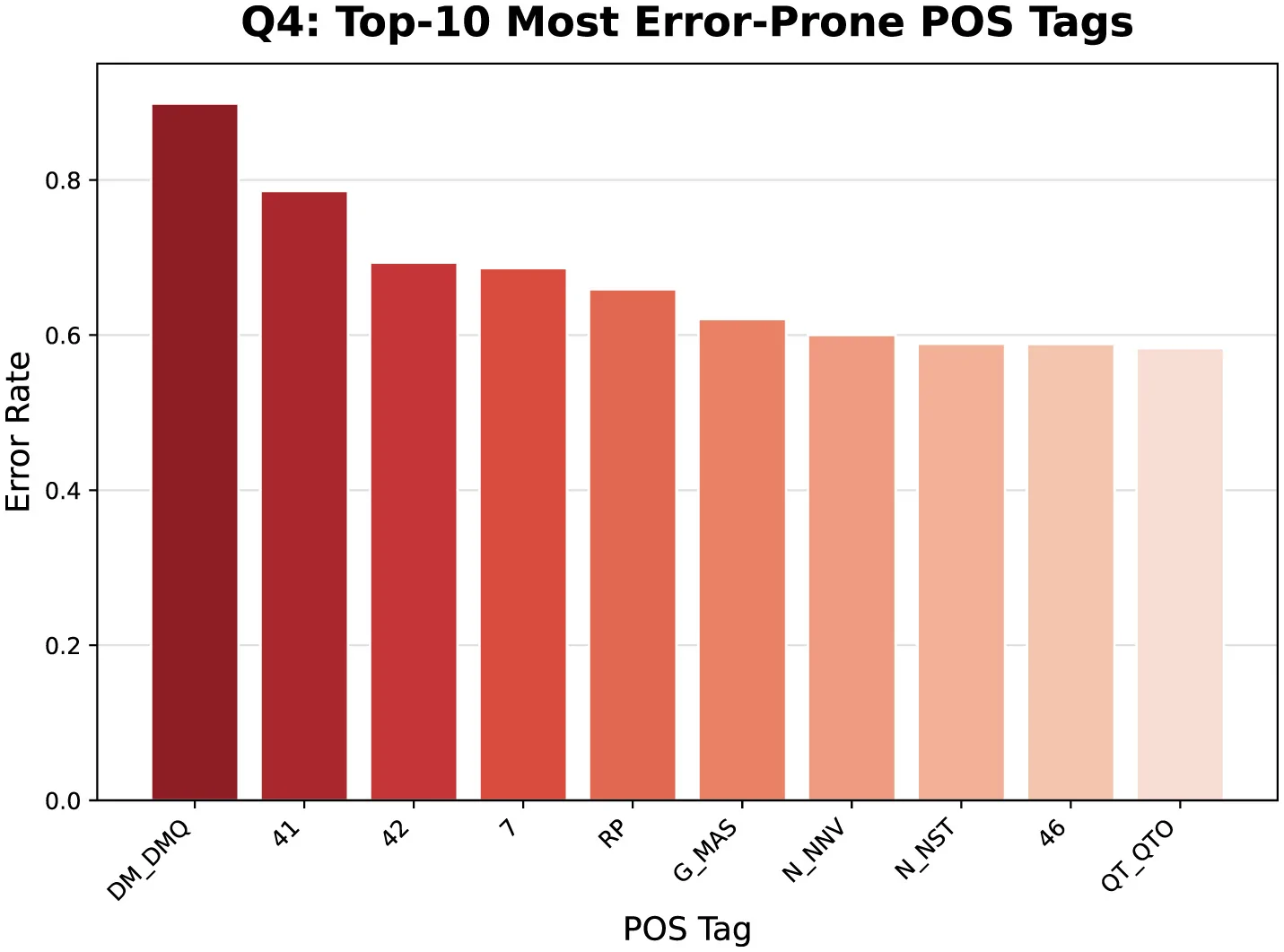
Distribution of the top 10 most frequently misclassified POS tags.

Figure 15 identifies the top-10 error-prone POS tags. The worst tag was DM_DMQ, with an error rate of 0.8986 in 69 instances. Other error-prone categories were tag class 41 with an error rate of 0.7859 across 2,083 instances, tag class 42 with 0.6934 across 1,448 instances, tag class 7 with 0.6864 across 118 instances, and RP with 0.6591 across 443 instances. The crucial point here is that the difficulty is not restricted only to extremely rare classes. Some classes with large support still exhibit high error rates, suggesting that ambiguity in tag boundaries or contextual similarity between categories remains a serious challenge even when more training examples are available. These findings indicate that data sparsity in rare POS categories such as DM_DMQ and RP remains the primary challenge across the evaluated models.

Figure 5 provides a complete comparison, bringing together Accuracy, Precision, Recall, macro F1 (seen classes) and weighted F1. *BiLSTM + CRF* had highest precision (0.9167), Recall (0.875), and macro F1 (seen classes) (0.8831). *CRF Max (With Gazetteer)* showed highest Accuracy (0.8949) and weighted F1 (0.8937). *CRF (No Lang)* ranked second (0.8170) with macro F1 (all classes) and led on balance for each class. No single model had top scores on every metric simultaneously, attesting the fact that the appropriate choice depends on the use case, as outlined in Table 7.

Figure 3 offers a per-class view of performance on the top-5 frequent POS tags, revealing a clear family-level specialization between tag classes. For the two numerically indexed categories (tag classes 15 and 16), only the transformer and BiLSTM-based models produced predictions: *Weighted MuRIL* achieved the highest scores of 0.8980 on class 15 and 0.8593 on class 16, followed by *mBERT (Optimized)* at 0.8822 and 0.8120 respectively, while *BiLSTM + CRF (Weighted)* hit 0.8631 and 0.7755 and *(FastText + BiLSTM + CRF)* reached 0.7999 and 0.6890. CRF-based models produce no predictions for these classes, indicating they collapse them into OTHER. For the three high-frequency complex structured categories N_NN, RD, and V_VM_VF, the pattern reverses entirely: CRF-based models lead the models, with *CRF Max (With Gazetteer)* reaching 0.9171 on N_NN, 0.9823 on RD, and 0.8501 on V_VM_VF, and *CRF Max (No Gazetteer)* achieving 0.9149, 0.9835, and 0.8476 on the same tags. Transformer and BiLSTM models produce no scores on these three categories in this figure. This mutual exclusivity confirms that model families are not simply better or worse overall but are specialized by tag type, and that no single architecture dominates uniformly across all five categories.

**Figure 16:**
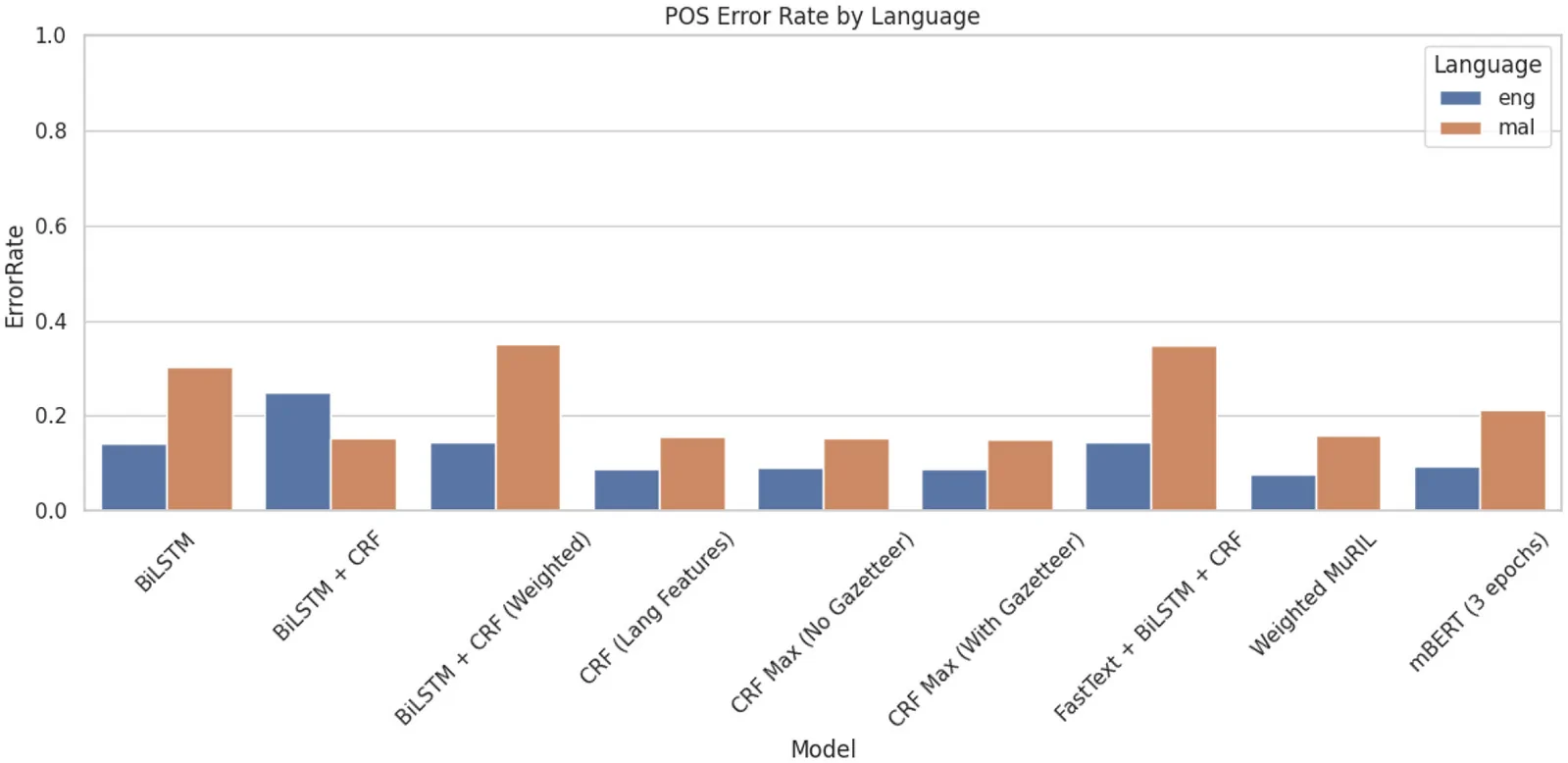
Comparison of POS error rates by language (English and Malayalam) across all models.

Figure 16 presents the POS error rate broken down by language (English vs Malayalam) for each model. Malayalam tokens consistently exhibited higher error rates than English tokens across most models, affirming the intense morphological complexity of Malayalam, besides the relative scarcity of Malayalam-specific pre-training in most architectures. *BiLSTM + CRF (Weighted)* and *(FastText + BiLSTM + CRF)* showed the largest Malayalam error rate at 0.35, while *Weighted MuRIL* achieved the lowest Malayalam error rate at 0.16. English error rates were generally lower and stable across models, ranging from approximately 0.07 to 0.25, with *BiLSTM + CRF* showing a notably higher English error rate (0.25) relative to Malayalam error rate of (0.15).

Figure 4 presents a comparison of micro F1 and macro F1, with highlights of how each model performed across common and less common classes. *CRF (No Lang)* had the smallest reported gap at 0.0466, which suggests the consistent behavior across class frequencies in this analysis. Other CRF variants also showed relatively controlled gaps in the range of (0.13–0.14). On the other hand, *BiLSTM + CRF* showed the largest reported micro–macro gap of 0.5056, with micro F1 of 0.8000 and macro F1 (all classes) of 0.2944. This gap arose because the macro F1 in Q3 is computed over the full tag set including every class, the (BiLSTM + CRF) model never predicted. The macro F1 (seen classes) of 0.8831 reported in Q6 were computed only over the classes predicted by the (BiLSTM + CRF) model.

### 5.4 Summary

The results discussed in the above section, demonstrate that no single model is best across all standard machine learning performance metrics. *BiLSTM + CRF* leads on macro F1 (seen classes) (0.8831), precision (0.9167), and recall (0.875), but its macro F1 (all classes) of 0.2944 and low accuracy of 0.800 reveal that its strength is concentrated on frequent tag classes. *CRF Max (With Gazetteer)* achieves the best accuracy (0.8949) and weighted F1 (0.8937), making it the strongest model for token-level correctness on dominant classes. *CRF (No Lang)* is the most balanced model overall, leading on macro F1 (all classes) (0.8170) and achieving the smallest micro–macro gap (0.0466), indicating consistent performance across both common and rare tag categories. Among transformer models, *mBERT (Optimized)* outperforms *Weighted MuRIL* on macro F1 (0.7464 vs. 0.7205) and accuracy (0.8517 vs. 0.8707^1^), while *Weighted MuRIL* achieves the best weighted F1 (0.8644) and the best per-class F1 on tag classes 15 and 16. Across all models, the POS categories that consistently pose the greatest challenge are *JJ*, *RB*, and V_VM_VNF_VBN. Error analysis confirms that the most problematic tags—DM_DMQ (error rate 0.8986), tag class 41 (0.7859), tag class 42 (0.6934), tag class 7 (0.6864), and *RP* (0.6591)—are predominantly low-frequency categories, with data sparsity as the primary bottleneck across all models (Figure 15).

## 6 Comparison with existing work

The results of this study can be placed in context by comparing with existing published approaches for code-mixed POS tagging on Indian Language pairs. While direct comparison is difficult due to differences in datasets, tag sets, and language pairs, broad trends can be observed.

For Hindi-English code-mixed POS tagging, systems participating in the ICON and EMNLP shared tasks, using CRF-based models with handcrafted features, have reported macro F1 scores ranging from approximately 0.72 to 0.85, which is consistent with the CRF results reported in this paper. Our Regularized CRF achieved a macro F1 of 0.7618, comparable to the best reported CRF systems for related language pairs.

The (BiLSTM + CRF) architecture used in this paper followed the design of Lample et al. (2016), which reported state-of-the-art NER performance using the same architecture. For POS tagging, similar (BiLSTM + CRF) systems have achieved macro F1 scores in the range of (0.85–0.92) on standard English benchmarks. Our result of 0.8831 for macro F1 (seen classes) on code-mixed data is notable given the additional complexity introduced by language switching.

For transformer-based approaches, Khanuja et al. (2020) reported that MuRIL outperformed mBERT on several Indian language classification and sequence labeling tasks. However, in our evaluation on English-Malayalam code-mixed POS tagging, *mBERT (Optimized)* achieved a macro F1 of 0.7464 compared to *Weighted MuRIL*'s macro F1 of 0.7205, meaning mBERT performed modestly better in this specific benchmark. Both models reported their weighted F1 as well: *mBERT (Optimized)* scored a weighted F1 of 0.8495 versus *Weighted MuRIL*'s weighted F1 score of 0.8644, showing that MuRIL is stronger on frequent classes when measured by weighted F1. The observed discrepancy in macro F1 may be ascribed to the specific nature of the code-mixed dataset, which may contain Romanized Malayalam forms that mBERT handled more effectively through its broader multilingual pre-training, or it may reflect the smaller sized fine-tuned dataset, to the detriment of larger specialized models like MuRIL.

A noteworthy finding that distinguishes this work from prior studies is the detailed micro–macro F1 gap analysis (Figure 4). The large gap for (BiLSTM + CRF) (0.5056), observed in the Q6 analysis, reveals that Accuracy can conceal extremely deficient performance on minority classes—a finding with practical implications for deployment of POS taggers in real-world code-mixed NLP pipelines, where unique grammatical categories may carry important semantic information.

The precision–recall gap analysis (Figure 5) also revealed an interesting split between model families. Models like (BiLSTM + CRF) (Weighted) and (FastText + BiLSTM + CRF) showed large precision–recall gaps of 0.1487 and 0.1146 respectively, indicative of their over-conservative feature. In contrast, mBERT (Optimized) showed the contrasting phenomena of Recall exceeding Precision by 0.0808,i.e, its over-predicting characteristic. The well-balanced models—BiLSTM (gap 0.0364), Regularized CRF (gap 0.0384), and CRF (Lang Features) (gap 0.0384)—showed Precision and Recall closest together, reflective of more reliable, generalizable prediction behavior.

Overall, this study provided the most comprehensive comparison of models slated for English-Malayalam code-mixed POS tagging published to date, covering 14 model configurations with detailed per-class and error-level analysis, beyond what has been reported in prior work on this language pair.

## 7 Conclusion and future work

### 7.1 Conclusion

This paper presented a comprehensive comparison of deep learning approaches for cross-linguistic Part-Of-Speech tagging, applied to English–Malayalam code-mixed data. We conducted performance analysis of 14 model configurations ranging from traditional models to transformer-based architectures, using standard machine learning performance metrics- accuracy, precision, recall, macro F1, weighted F1, Confusion Matrices.

The key findings are as follows. No single model dominates across all metrics. *BiLSTM + CRF* achieves the best macro F1 (seen classes) (0.8831), precision (0.9167), and recall (0.875), but its macro F1 (all classes) of 0.2944 and accuracy of 0.800 indicate that its gains are concentrated on frequent tag classes. *CRF Max (With Gazetteer)* leads on accuracy (0.8949) and weighted F1 (0.8937). *CRF (No Lang)* is the most robust model overall, achieving the best macro F1 (all classes) (0.8170), second-best macro F1 (seen classes), and the smallest micro–macro gap (0.0466). Among transformer models, *mBERT (Optimized)* (macro F1 0.7464, weighted F1 0.8495) achieves higher macro F1 than *Weighted MuRIL* (macro F1 0.7205), while *Weighted MuRIL* achieves the higher weighted F1 (0.8644) and the best per-class scores on tag classes 15 and 16. The addition of LID features and gazetteer resources to CRF models did not yield significant improvements. Adjectives (*JJ*), adverbs, and rare categories like DM_DMQ consistently pose the greatest challenge across all models, with data sparsity confirmed as the primary bottleneck. The micro–macro F1 gap analysis reveals that high overall accuracy can coexist with poor minority class coverage, which has important implications for real-world deployment.

### 7.2 Future work

Several directions for future work arise from this study. First, data augmentation techniques such as back-translation, synonym substitution using multilingual embeddings, or synthetic code-mixing could help address the severe class imbalance observed for rare POS categories. Second, language-aware fine-tuning strategies for MuRIL such as using a curriculum that progressively introduces code-mixed examples may help close the performance gap with mBERT. Third, ensemble approaches that combine the precision strength of (BiLSTM + CRF) with the recall strength of transformer-based models are worth exploring. Fourth, extending the annotated dataset with more samples from diverse social media domains could improve generalizability. Future releases of this dataset would benefit from multi-annotator validation to establish formal inter-annotator agreement, which was not computed for the current version. Finally, investigating cross-lingual transfer from better-resourced language pairs such as Hindi–English to Malayalam–English could provide useful inductive biases for the tagging model, also, employing FastText vectors trained specifically on Malayalam or code-mixed corpora, which this study did not evaluate, remains an open direction and replicating all 14 configurations across multiple random seeds to report variance and confidence intervals, which the current single-run design does not capture. A notable and unanticipated finding was that pretrained multilingual transformers did not translate their Malayalam pretraining into a decisive advantage over models trained solely on the target code-mixed dataset–CRF (No Lang) and (BiLSTM + CRF) matched or exceeded mBERT and MuRIL on macro F1 and precision/recall respectively. This suggests that generic multilingual pretraining may not fully capture the specific lexical and grammatical patterns of English–Malayalam code-mixed text. Future work should therefore investigate pretraining or fine-tuning strategies that expose transformer models directly to code-mixed data, rather than relying solely on monolingual-per-language pretraining. In addition, this limitation motivates the evaluation of Malayalam-specific and multilingual pretrained embeddings, such as multilingual FastText, XLM-R, and mBERT-based embeddings, to better understand the impact of embedding initialization on English–Malayalam code-mixed POS tagging performance.

## Data Availability

The raw data supporting the conclusions of this article will be made available by the authors, without undue reservation.

## Footnotes

1 Weighted MuRIL achieves higher raw accuracy but lower macro F1 (seen classes).
