# Supplementary material for: Study of deep learning cues for cross linguistic part of speech tagging in English– Malayalam code-mixed data
Source: Front Big Data. 2026 Aug 28;9:1888885. doi: 10.3389/fdata.2026.1888885 (PMC13561951; doi:10.3389/fdata.2026.1888885)
Supplement: Supplementary file 1 [file Data_Sheet_1.pdf]

0.1 Figures

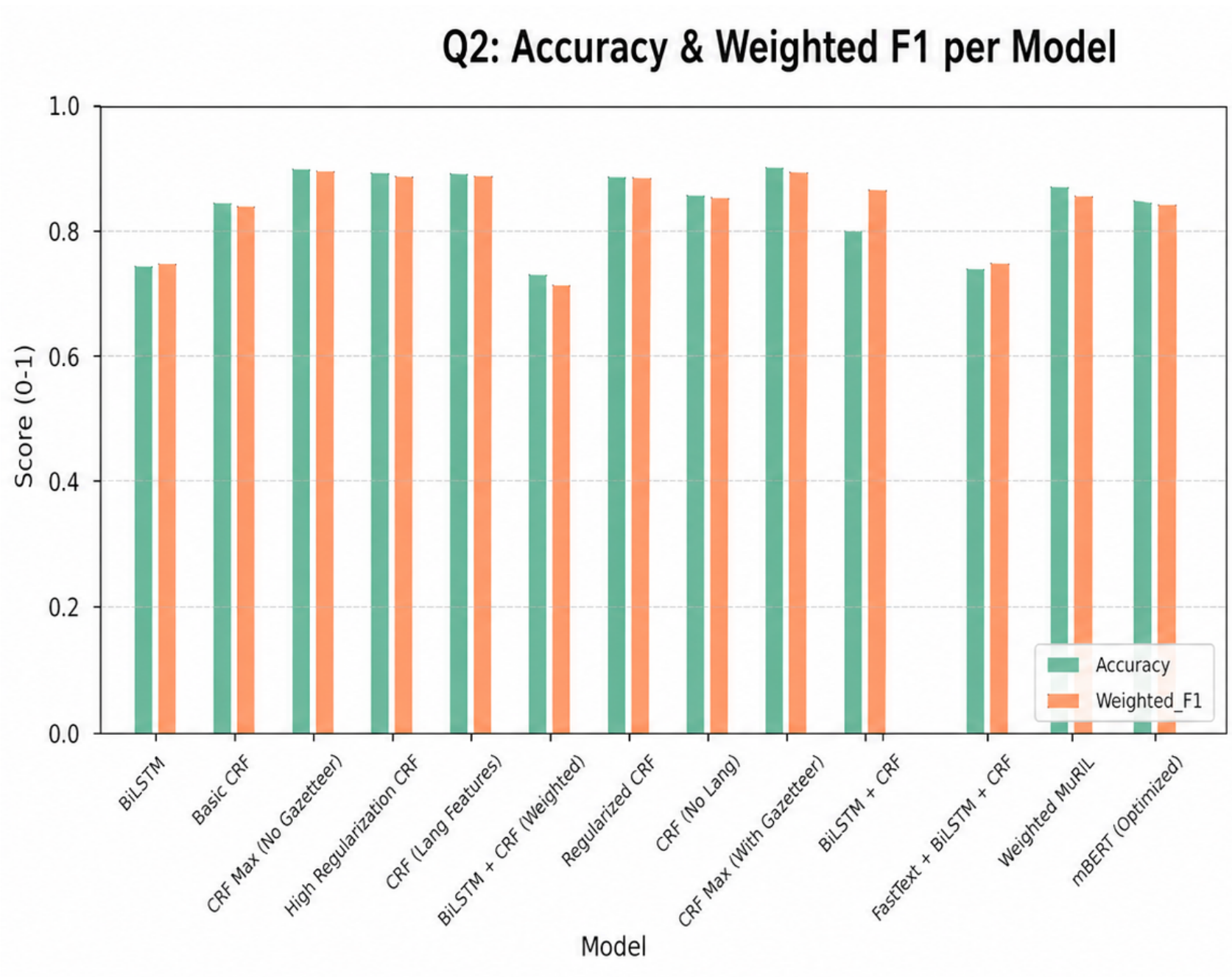

Model	Accuracy	Weighted_F1
BiLSTM	0.7527	0.7530
Basic CRF	0.8440	0.8406
CRF Max (No Gazetteer)	0.8938	0.8925
High Regularization CRF	0.8926	0.8913
CRF (Lang Features)	0.8927	0.8914
BiLSTM + CRF (Weighted)	0.7310	0.7197
Regularized CRF	0.8927	0.8914
CRF (No Lang)	0.8636	0.8603
CRF Max (With Gazetteer)	0.8949	0.8937
BiLSTM + CRF	0.8000	0.867532
FastText + BiLSTM + CRF	0.7366	0.7440
Weighted MuRIL	0.8707	0.8644
mBERT (Optimized)	0.8517	0.8495

Figure S1. Accuracy and weighted F1 comparison across models.

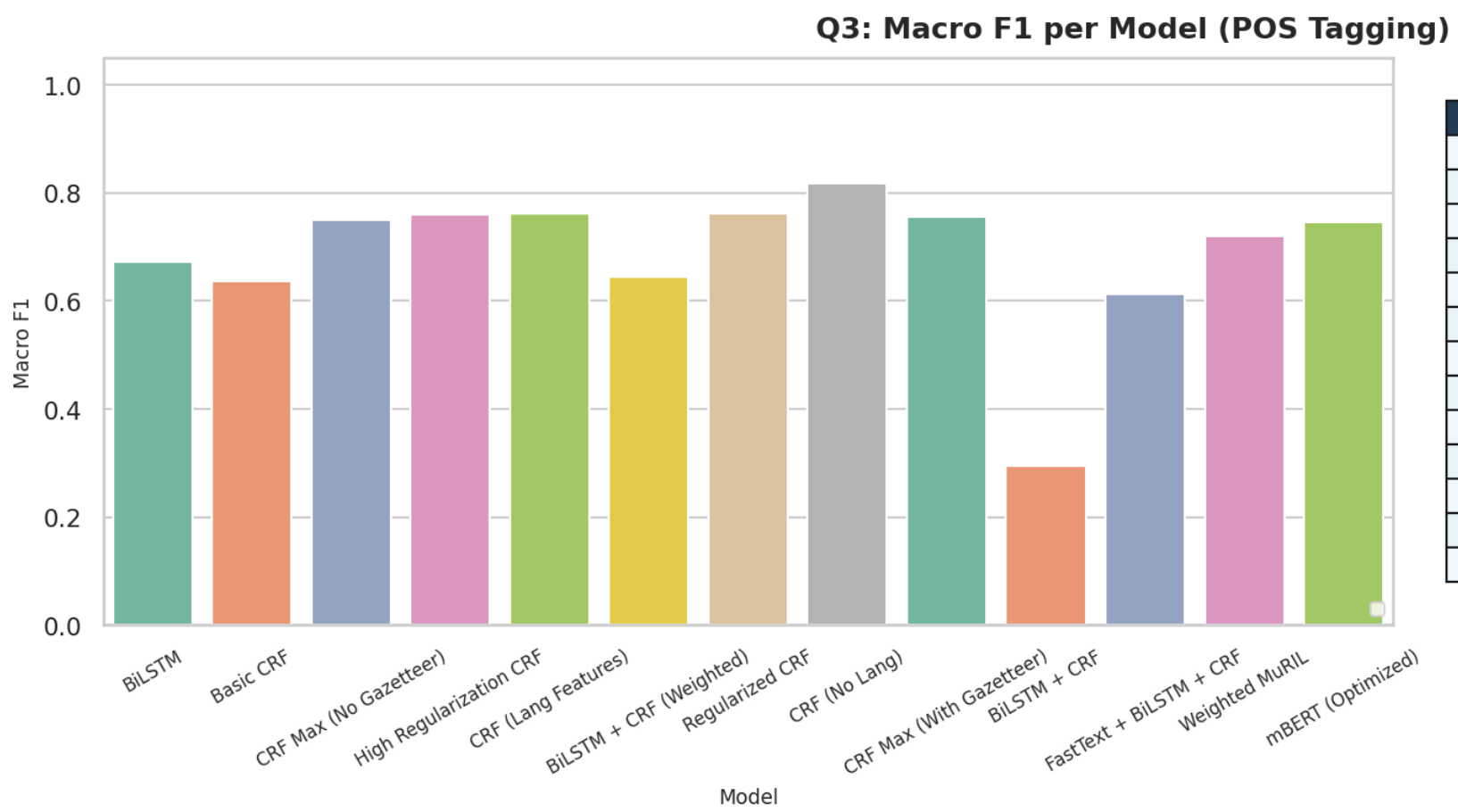

Model	Macro_F1
BiLSTM	0.6723
Basic CRF	0.6370
CRF Max (No Gazetteer)	0.7498
High Regularization CRF	0.7606
CRF (Lang Features)	0.7618
BiLSTM + CRF (Weighted)	0.6445
Regularized CRF	0.7618
CRF (No Lang)	0.8170
CRF Max (With Gazetteer)	0.7550
BiLSTM + CRF	0.2944
FastText + BiLSTM + CRF	0.6138
Weighted MuRIL	0.7205
mBERT (Optimized)	0.7464

Figure S2. Macro F1 (all classes) across all models.

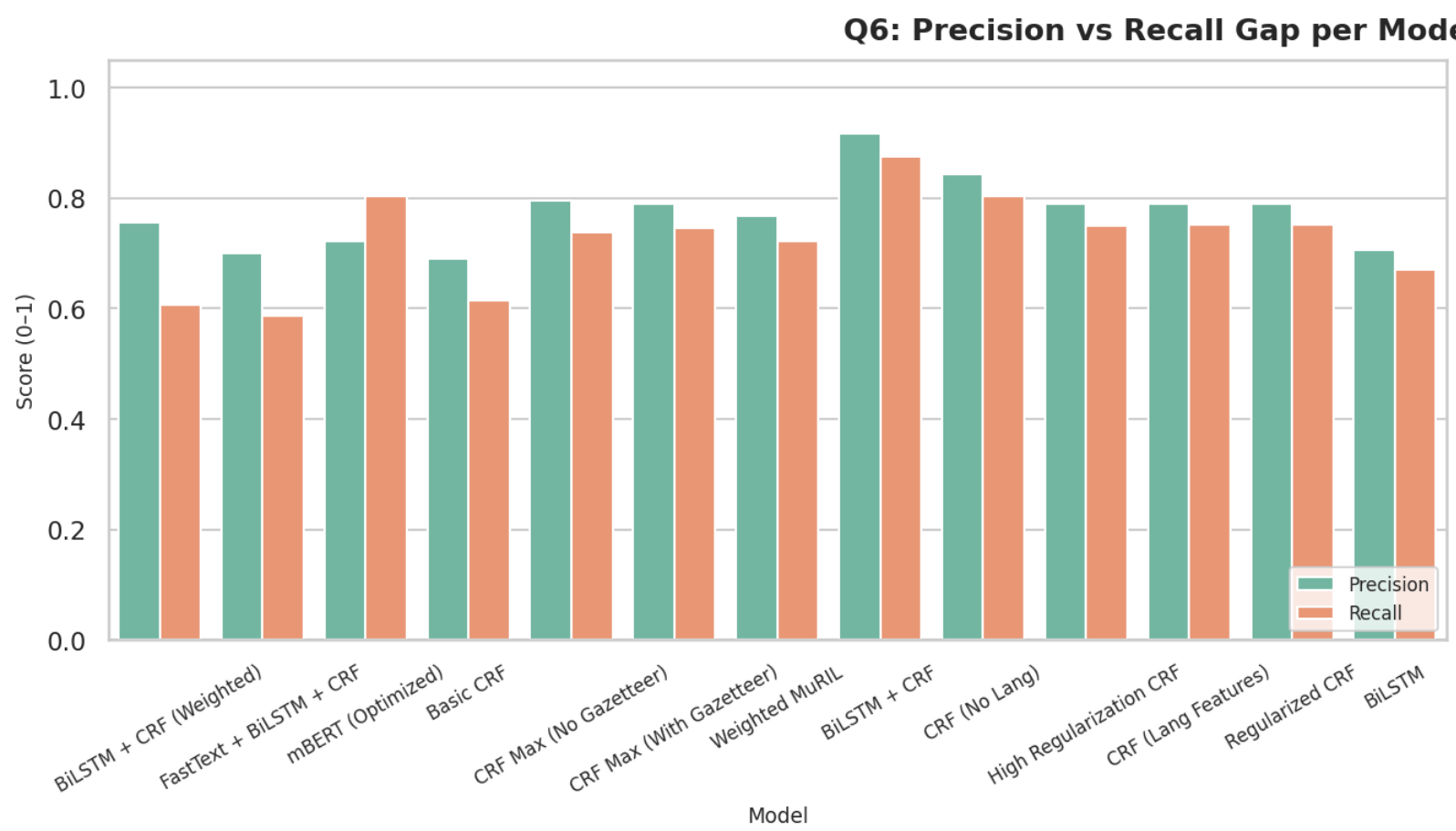

Model	Precision	Recall	P-R Gap
BiLSTM + CRF (Weighted	0.7558	0.6072	0.1487
FastText + BiLSTM + CRF	0.7008	0.5862	0.1146
mBERT (Optimized)	0.7220	0.8028	0.0808
Basic CRF	0.6899	0.6156	0.0743
CRF Max (No Gazetteer)	0.7962	0.7390	0.0572
CRF Max (With Gazetteer	0.7903	0.7453	0.0450
Weighted MuRIL	0.7672	0.7231	0.0441
BiLSTM + CRF	0.9167	0.8750	0.0417
CRF (No Lang)	0.8443	0.8034	0.0409
High Regularization CRF	0.7904	0.7502	0.0402
CRF (Lang Features)	0.7903	0.7518	0.0384
Regularized CRF	0.7903	0.7518	0.0384
BiLSTM	0.7066	0.6702	0.0364

Figure S3. Precision-Recall Gap Across Evaluated Models.

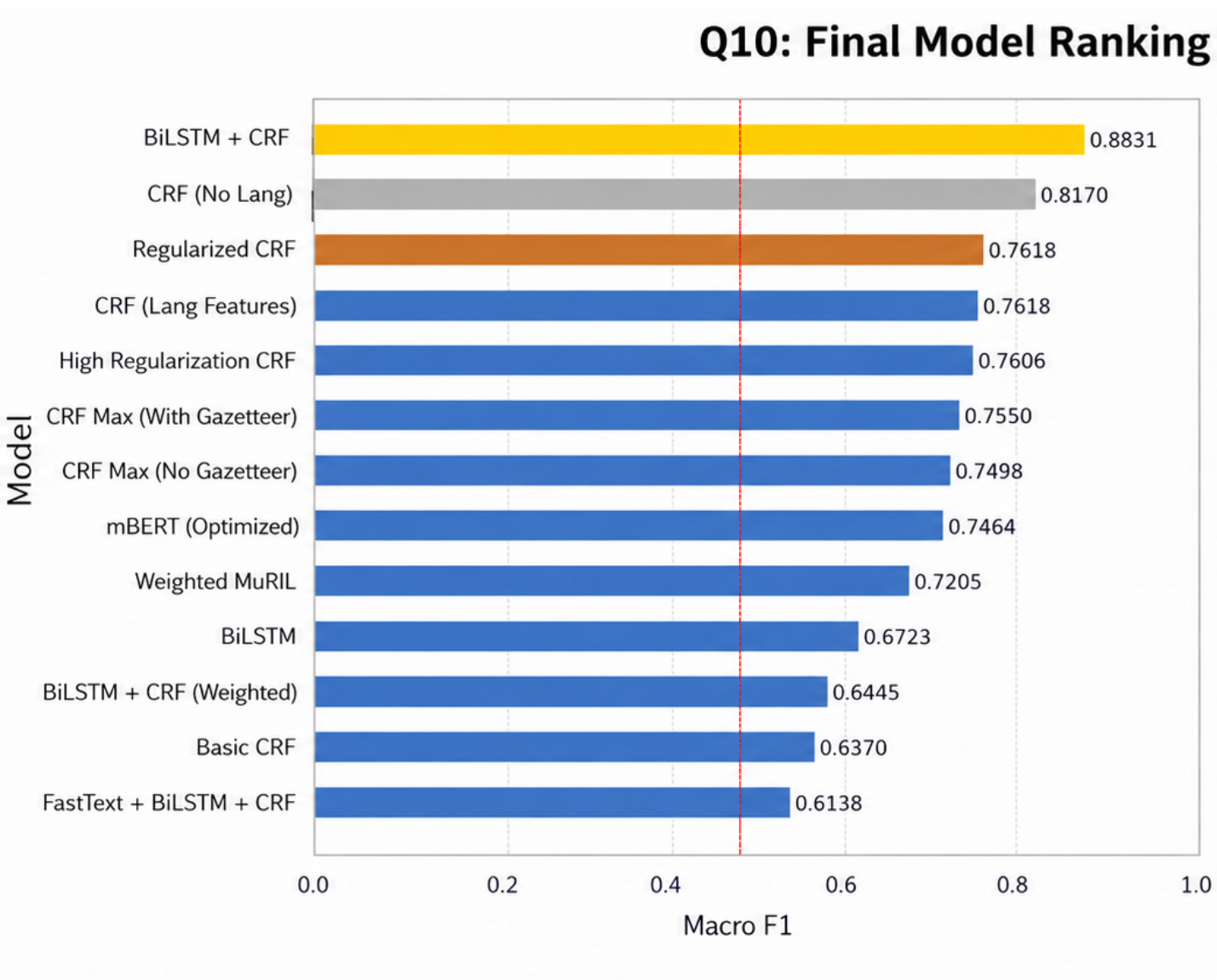

Rank	Model	Accuracy	Macro_F1	Weighted_F1	Precision	Recall
1	BiLSTM + CRF	0.8000	0.8831	0.867532	0.9167	0.8750
2	CRF (No Lang)	0.8636	0.8170	0.8603	0.8443	0.8034
3	Regularized CRF	0.8927	0.7618	0.8914	0.7903	0.7518
4	CRF (Lang Features)	0.8927	0.7618	0.8914	0.7903	0.7518
5	High Regularization CRF	0.8926	0.7606	0.8913	0.7504	0.7502
6	CRF Max (With Gazetteer)	0.8949	0.7550	0.8937	0.7403	0.7453
7	CRF Max (No Gazetteer)	0.8938	0.7498	0.8925	0.7962	0.7390
8	mBERT (Optimized)	0.8517	0.7464	0.8495	0.7220	0.8028
9	Weighted MuRIL	0.8707	0.7205	0.8644	0.7672	0.7231
10	BiLSTM	0.7527	0.6723	0.7530	0.7066	0.6702
11	BiLSTM + CRF (Weighted)	0.7310	0.6445	0.7197	0.7558	0.6072
12	Basic CRF	0.8440	0.6370	0.8406	0.6899	0.6156
13	FastText + BiLSTM + CRF	0.7366	0.6138	0.7440	0.7008	0.5862

Figure S4. Comparison of Model by Macro F1 (Seen Classes).

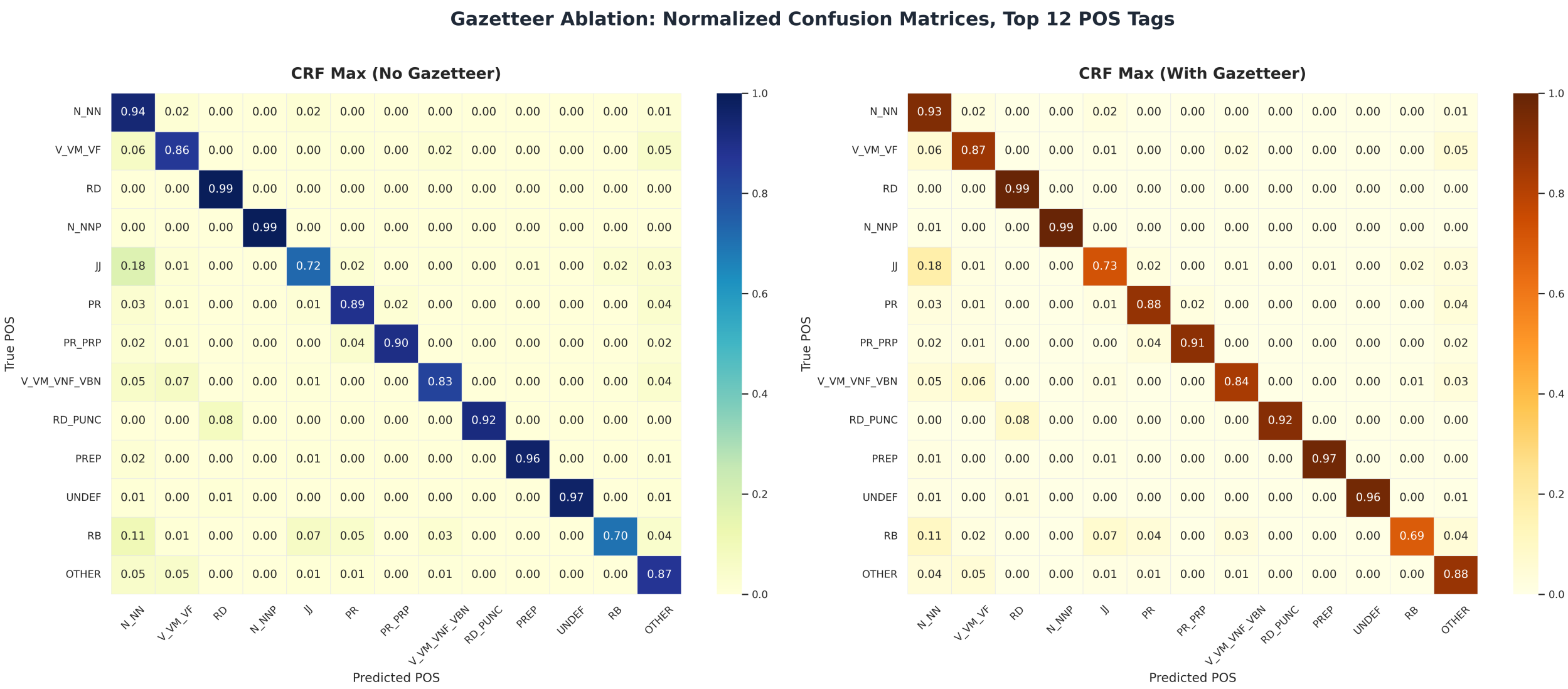

Figure S5. Evaluation of Max-CRF Models:With vs. Without Gazetteer Integration
